# The CsHSP17.2 molecular chaperone is essential for thermotolerance in *Camellia sinensis*

**DOI:** 10.1038/s41598-017-01407-x

**Published:** 2017-04-27

**Authors:** Mingle Wang, Zhongwei Zou, Qinghui Li, Kang Sun, Xuan Chen, Xinghui Li

**Affiliations:** 1 Tea Research Institute, Nanjing Agricultural University, Nanjing, 210095 China; 2 Department of Plant Science, University of Manitoba, Winnipeg, MB R3T 2N2 Canada

## Abstract

Small heat shock proteins (sHSPs) play important roles in responses to heat stress. However, the functions of sHSPs in tea plants (*Camellia sinensis*) remain uncharacterized. A novel *sHSP* gene, designated *CsHSP17.2*, was isolated from tea plants. Subcellular localization analyses indicated that the CsHSP17.2 protein was present in the cytosol and the nucleus. *CsHSP17.2* expression was significantly up-regulated by heat stress but was unaffected by low temperature. The *CsHSP17.2* transcript levels increased following salt and polyethylene glycol 6000 treatments but decreased in the presence of abscisic acid. The molecular chaperone activity of CsHSP17.2 was demonstrated *in vitro*. Transgenic *Escherichia coli* and *Pichia pastoris* expressing *CsHSP17.2* exhibited enhanced thermotolerance. The transgenic *Arabidopsis thaliana* exhibited higher maximum photochemical efficiencies, greater soluble protein proline contents, higher germination rates and higher hypocotyl elongation length than the wild-type controls. The expression levels of several HS-responsive genes increased in transgenic *A. thaliana* plants. Additionally, the *CsHSP17.2* promoter is highly responsive to high-temperature stress in *A. thaliana*. Our results suggest that CsHSP17.2 may act as a molecular chaperone to mediate heat tolerance by maintaining maximum photochemical efficiency and protein synthesis, enhancing the scavenging of reactive oxygen species and inducing the expression of HS-responsive genes.

## Introduction

Heat shock (HS) is induced by a higher temperature, which is approximately 10–15 °C above the optimal growth temperature^1, 2^. After exposure to HS, prokaryotic and eukaryotic cells produce a group of heat shock proteins (HSPs)^2, 3^. The HSPs can be divided into the following five categories based on their approximate molecular weights: HSP100, HSP90, HSP70, HSP60, and small HSPs (sHSPs). The sHSPs are the most prevalent in plants, and their importance is implied by their unusual abundance and diversity^4^. Six classes of sHSPs have been identified based on their amino acid sequence similarities, immunological cross-reactivities, and intracellular localizations^4, 5^. Members of Classes I and II are localized in the cytosol and the nucleus. Classes III, IV, V, and VI consist of sHSPs present in the chloroplast, endoplasmic reticulum, mitochondrion, and membrane, respectively^6, 7^.

Molecular chaperones are responsible for protein folding, assembly, translocation and degradation in many normal cellular processes. They also stabilize proteins and membranes, and assist in protein refolding under stress conditions^4^. Previous studies revealed that a wide range of sHSPs have molecular chaperone activities *in vitro* and *in vivo* ^8, 9^. Analyses of sHSP transcript levels indicated that some sHSPs were undetectable in the absence of stress, but rapidly accumulated following exposure to HS^10, 11^. In addition to HS, the expression of *sHSP* genes can be induced by other abiotic stress, including salinity^12^, low temperature^13^ and drought^14^. Furthermore, some plant sHSPs are only produced in specific tissues or during particular developmental stages^10, 15^. Previous studies have demonstrated that heterologous expression of *sHSP* genes confers abiotic stress tolerance in transgenic *A. thaliana* ^16^, rice^17^, tomato^18^, potato^19^, and tobacco^20^, suggesting that sHSPs are involved in stress tolerance. Ruibal *et al*.^21^ found that a functional sHSP *PpHsp16.4* was essential for recovery from heat, salt and osmotic stress in *Physcomitrella patens*. However, overexpression of *AsHSP17*, a new *sHSP* gene from creeping bentgrass (*Agrostis stolonifera*), negatively regulate plant responses to adverse environmental stresses in transgenic *A. thaliana* ^22^. Recently, OsHSP18.2, a sHSP from rice (*Oryza sativa*), was shown to play important roles in seed vigor, longevity and seedling establishment in transgenic *A. thaliana* ^23^.

Tea plants [*Camellia sinensis* (L.) O. Kuntze] are important leaf-type woody crops used for the production of non-alcoholic beverages worldwide. The plants must cope with various abiotic stress during their lifecycle^24^, including low and high temperatures^25^, drought^26^, and salinity^27^. High-temperatures are likely the most important abiotic stress for tea plants because they can considerably affect crop quality and yield. Thus, it is important to understand the molecular mechanisms regulating heat tolerance in *C. sinensis*. We previously isolated several HS-inducible genes from tea plants using a suppression subtractive hybridization method (data not shown). Pre-experimental results revealed that *CsHSP17.2* (GenBank accession number: KU244518) was more highly expressed following exposure to HS than the other HS-inducible genes. These results suggested that *CsHSP17.2* affected heat tolerance in *C. sinensis*. However, the exact relationship between *CsHSP17.2* and thermotolerance has not been fully characterized. In the present study, we analyzed the CsHSP17.2 functions associated with thermotolerance by overexpressing *CsHSP17.2* in *E. coli* (a prokaryote), *P. pastoris* and *A. thaliana* (eukaryotes). Furthermore, we report the cloning of its promoter by genome walking and its characterization using transgenic approaches. The results of this study may lead to a more thorough characterization of the molecular basis of thermotolerance in *C. sinensis*.

## Results

### Isolation and sequence analysis of *CsHSP17.2* gene

The full-length cDNA of *CsHSP17.2* gene consisted of 811 bp, with a 453-bp open reading frame (ORF) encoding a 150-amino-acid protein (predicted molecular mass of 17.2 kDa). Sequence alignments with the deduced CsHSP17.2 protein sequence and those of other plant sHSPs indicated that CsHSP17.2 contains a conserved 90-amino-acid C-terminal α-crystallin domain^5^, which can be further divided into two homologous regions, namely, consensus region I (positions +110 to +135) and consensus region II (positions +59 to +82) (Fig. S1a). A phylogenetic analysis revealed that CsHSP17.2 was highly similar to Class I sHSPs and was closely related to proteins from *Gossypium hirsutum* GhHSP17.9 (AEH30706) and *Jatropha curcas* JcHSP17.3 (XP_012066921) (Fig. S1b).

### Subcellular localization of CsHSP17.2 protein

**Figure 1:**
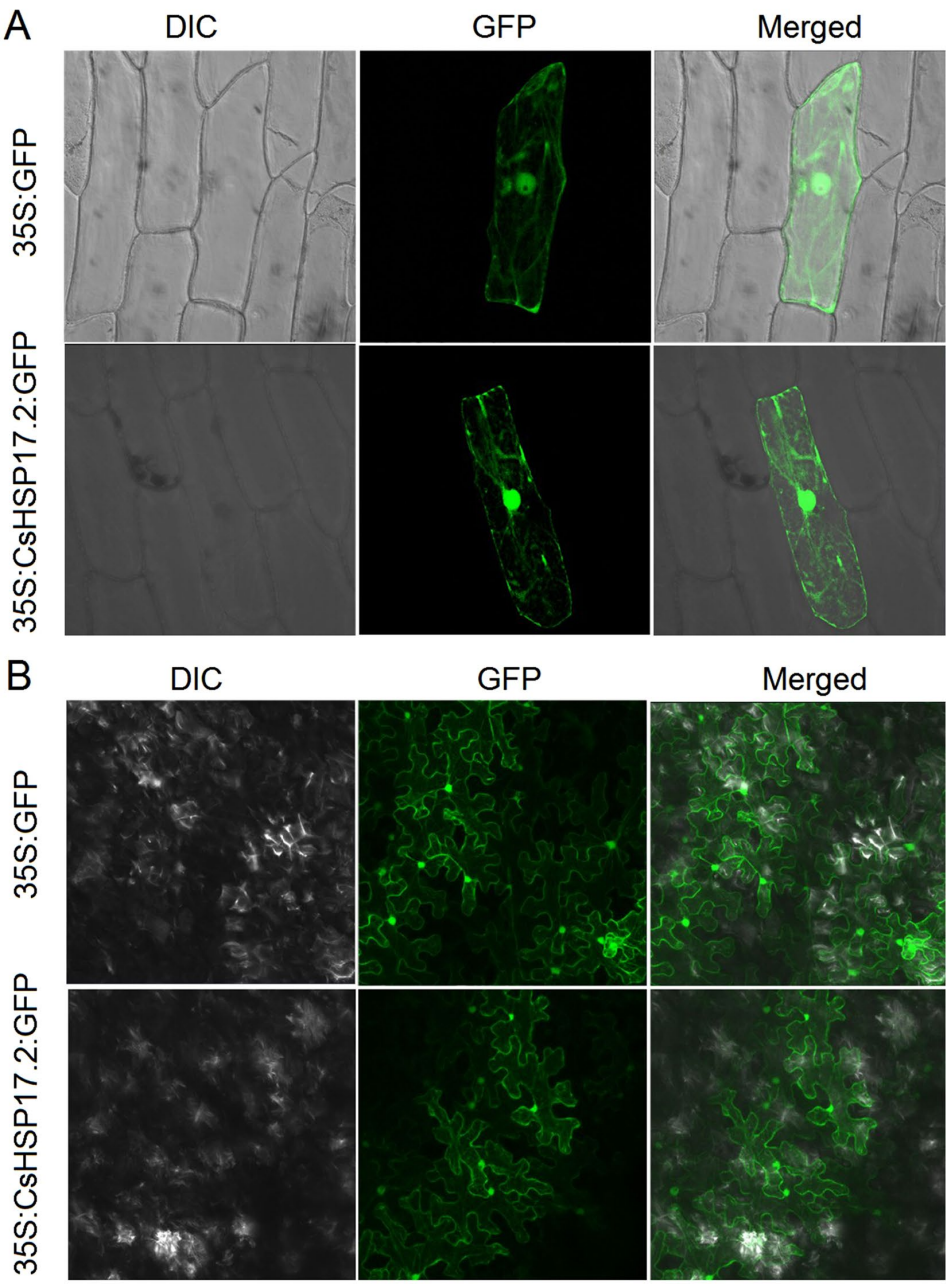
CsHSP17.2 protein accumulates in the nucleus and the cytosol. (**A**) Subcellular localization of CsHSP17.2 in onion (*Allium cepa*) epidermal cells. (**B**) Subcellular localization of CsHSP17.2 in tobacco (*Nicotiana benthamiana*) leaves. 35S:GFP indicates the vector control, and 35S:CsHSP17.2:GFP refers to the target protein.

To clarify the CsHSP17.2 biological functions, subcellular localization investigations were completed using onion epidermal cells and tobacco leaves producing the CsHSP17.2:GFP fusion protein. The 35S:GFP signals were distributed throughout the cytosol and the nucleus in onion (Fig. 1A) and tobacco epidermal cells (Fig. 1B). As predicted by the protein subcellular localization prediction software WoLF PSORT^28^, diffuse 35S:CsHSP17.2:GFP signals were detected in the cytosol and nucleus of onion (Fig. 1A) and tobacco epidermal cells (Fig. 1B).

### *CsHSP17.2* transcription profiles under heat shock (HS) and recovery conditions

**Figure 2:**
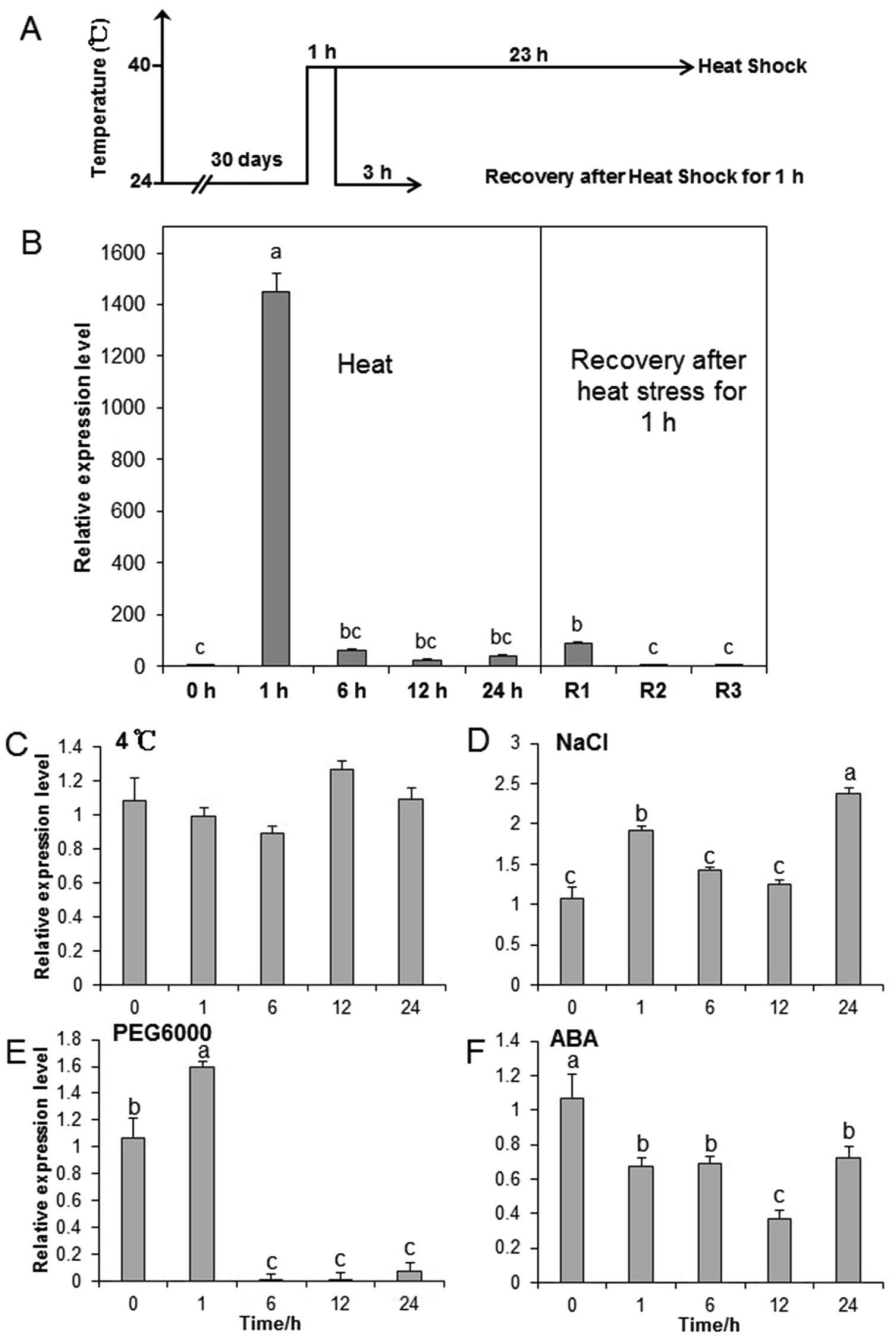
Transcription profiles of *CsHSP17.2* gene under abiotic stress. (**A**) Schematic diagram of heat stress and recovery. (**B**) *CsHSP17.2* expression levels under heat stress and recovery. R1, R2 and R3 indicate recovery from heat shock (HS) for 1, 2, and 3 h, respectively. Tea plants exposed to low temperature (4 °C) (**C**), 300 mM NaCl (**D**), 20% (w/v) PEG6000 (**E**) and 50 µM ABA (**F**). Different letters indicate significant differences (*P* < 0.05) between treatments and the control (0 h).

To determine whether *CsHSP17.2* expression under HS conditions is time-dependent, tea plants were incubated at high temperatures (40 °C) for different times (Fig. 2A). The abundance of *CsHSP17.2* transcripts in plants exposed to HS initially increased very quickly, with the highest levels (approximately 1,450-fold higher than pre-treatment levels) occurring within 1 h. Transcript levels significantly decreased after 6 h (*P* < 0.01). Following the HS treatment for 1 h, the tea plants were returned to normal conditions to recover from the heat stress. The *CsHSP17.2* transcript levels decreased significantly within 1 h of recovery and were almost undetectable after 3 h (*P* < 0.01) (Fig. 2B).

### Analysis of *CsHSP17.2* expression following exposure to cold, salinity and exogenous abscisic acid (ABA)

To investigate whether the *CsHSP17.2* transcript levels were regulated by other abiotic stress, tea plants were exposed to various treatments. Under cold stress conditions, there were no significant changes in *CsHSP17.2* expression levels within the first 24 h (Fig. 2C). The abundance of *CsHSP17.2* transcripts in salt-stressed plants increased within 1 h of treatment, decreased significantly after 6 and 12 h (*P* < 0.05), and then peaked at 24 h (Fig. 2D). When exposed to drought stress, *CsHSP17.2* transcript levels were high at 1 h and then decreased significantly after 6 h (*P* < 0.05) (Fig. 2E). Finally, exogenous ABA treatment significantly inhibited the *CsHSP17.2* transcription levels in tea plants (*P* < 0.05) (Fig. 2F). These results indicated that *CsHSP17.2* influenced drought and salinity stress responses and ABA signal transduction pathways.

### *CsHSP17.2* overexpression in *E. coli* cells

**Figure 3:**
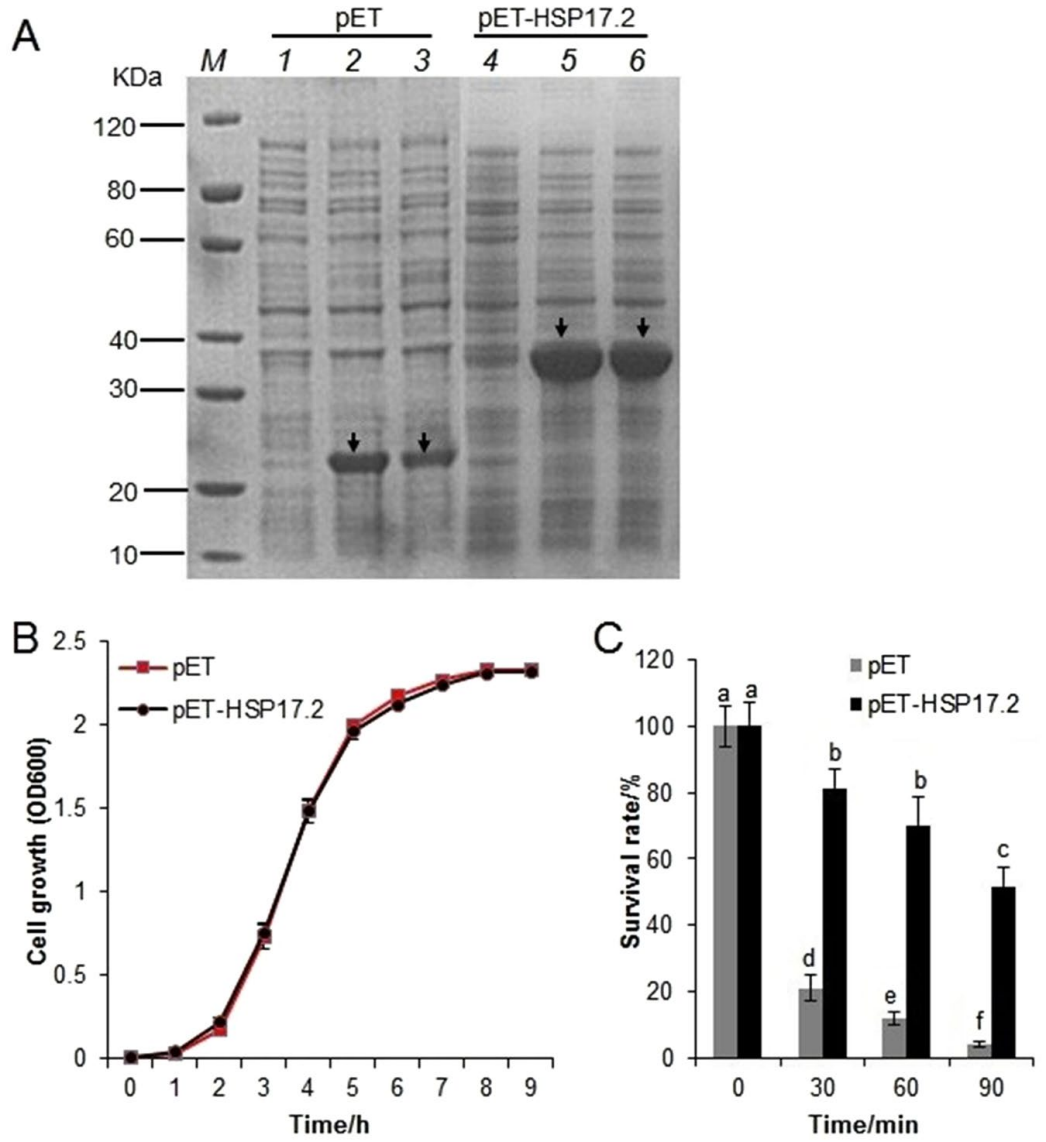
Heterologous expression of *CsHSP17.2* in *E. coli*. (**A**) Expression of the CsHSP17.2 protein in *E. coli* assessed by SDS-PAGE analysis. *M*, protein molecular weight marker; lane 1: whole cell lysate of un-induced cells containing the empty vector pET; lane 2: whole cell lysate of cells cntaining the empty vector and induced by 0.2 mM IPTG at 15 °C for 16 h; lane 3: whole cell lysate of cells containing the empty vector and induced by 0.2 mM IPTG at 37 °C for 4 h; lane 4: whole cell lysate of un-induced cells containing pET-HSP17.2; lane 5: whole cell lysate of cells containing pET-HSP17.2 and induced by 0.2 mM IPTG at 15 °C for 16 h; lane 6: whole cell lysate of cells containing pET-HSP17.2 and induced by 0.2 mM IPTG at 37 °C for 4 h. The induced proteins are indicated by *black arrows*. (**B**) Growth analysis of transformed *E. coli* BL21 (DE3) cells under normal conditions. (**C**) Protective effect of recombinant CsHSP17.2 on cell viability during HS *in vivo*. The mean values of three independent experiments are plotted with error bars indicating standard deviations. The significant differences are indicated by different letters (*P* < 0.05).

We completed reverse transcription polymerase chain reaction (RT-PCR) experiments to ensure that appropriate transgenic *E. coli* cells had been generated. The resulting amplicons were 202 and 655 bp long, which corresponded to the control and *CsHSP17.2*-overexpressing lines, respectively (Fig. S2a). The bands corresponding to the expected 22-kDa thioredoxin/histidine tag were larger in the control *E. coli* lines (i.e., those harboring the empty vector) treated with 0.2 mM isopropyl-β-D-thiogalac-topyranoside (IPTG) than in the non-induced control *E. coli* lines. Additionally, an approximately 36-kDa band was detected for *CsHSP17.2*-overexpressing lines induced with IPTG, which matched the expected size of the CsHSP17.2 protein fused to the thioredoxin/histidine tag (Fig. 3A).

Under the premise that the pET *E. coli* overexpression system works normally, we performed cell viability assays to investigate the possible functions of CsHSP17.2. There was no obvious difference in the growth rates of the pET and pET-HSP17.2 strains, indicating that *CsHSP17.2* overexpression did not affect *E. coli* growth under normal conditions (Fig. 3B). Under heat stresses, the *E. coli* survival decreased rapidly, but the *CsHSP17.2*-overexpressing cells were more viable than the control cells (*P* < 0.01). After a 90-min heat treatment, more than 95% of the pET cells died, whereas approximately 51% of pET-HSP17.2 cells survived (Fig. 3C). This observation suggested that CsHSP17.2 increased the thermotolerance of the transgenic *E. coli* cells.

### Constitutive expression of *CsHSP17.2* confers thermotolerance in transgenic *P. pastoris*

**Figure 4:**
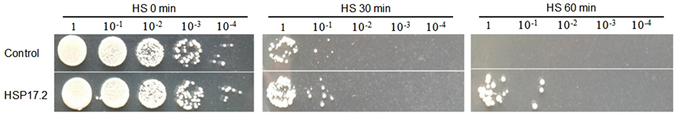
Heterologous expression of *CsHSP17.2* in *P. pastoris* under heat shock (HS). Control indicates the transformant harboring the empty vector pPIC3.5 K, and HSP17.2 indicates the *P. pastoris* strains with recombinant plasmid pPIC3.5K-HSP17.2.

To confirm that CsHSP17.2 influenced thermotolerance, we examined the expression of *CsHSP17.2* in a eukaryotic organism. First, RT-PCR was used to verify that the recombinant plasmids were present in the transformed *P. pastoris* cells. The amplified fragments were 221 and 548 bp long, which were the expected sizes for the control and *CsHSP17.2-*overexpressing lines, respectively (Fig. S2b). The positive strains were used for subsequent thermotolerance assays. Similar growth rates were observed between the control and the *CsHSP17.2-*overexpressing lines at 30 °C. However, the growth rates were higher for the *CsHSP17.2-*overexpressing strains than the control after HS treatment for 30 or 60 min (Fig. 4). These results indicated that the constitutive expression of *CsHSP17.2* confers thermotolerance in *P. pastoris*.

### CsHSP17.2 exhibits molecular chaperone activity *in vitro*

**Figure 5:**
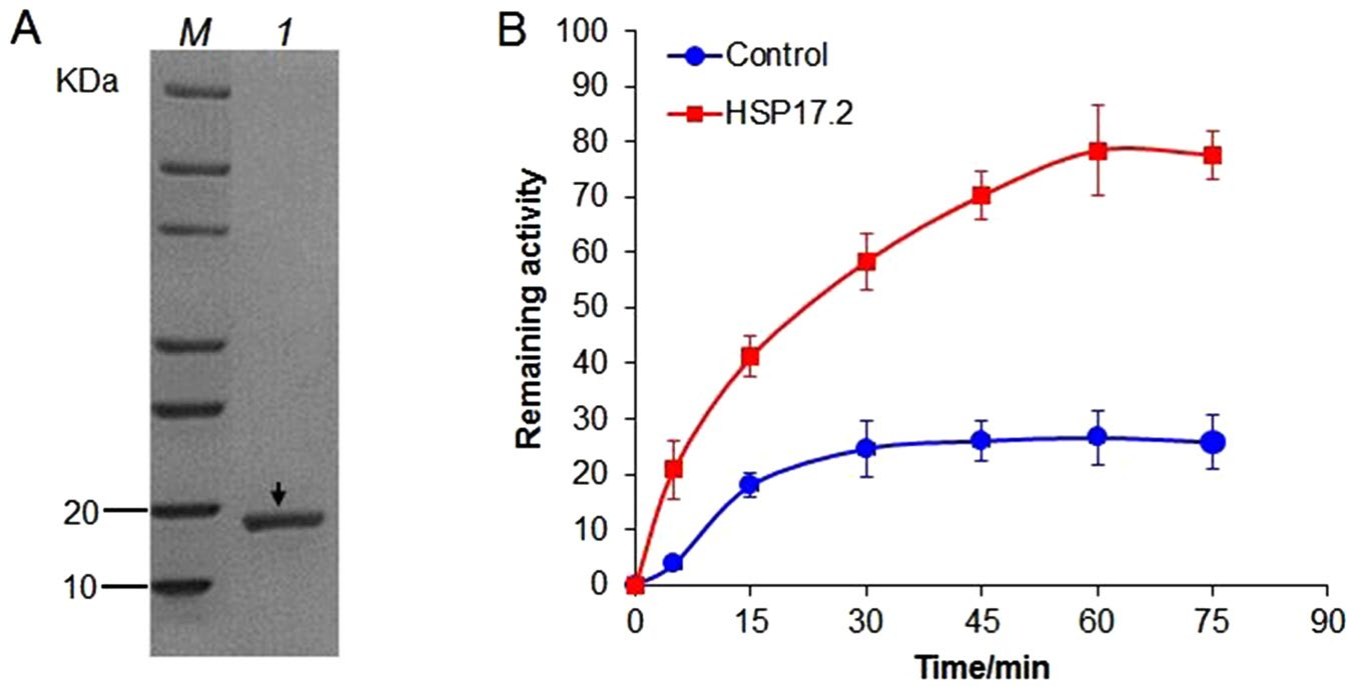
Purified recombinant CsHSP17.2 protein was separated on a 4–22% native gel (**A**) and the effect of CsHSP17.2 protein on the renaturation of chemically denatured citrate synthase (CS) (**B**). *M*, protein marker; lane 1: recombinant CsHSP17.2 protein. CS (15 μM) was denatured in 6 M guanidine hydrochloride for 2 h and then placed under refolding conditions containing 150 nM CsHSP17.2 proteins or H_2_O (Control). The remaining activity of CS is presented as the percentage of non-denatured CS activity. Error bars in figures represent standard errors generated from at least three replicate trials.

After the thioredoxin/hi-stidine-tagged CsHSP17.2 protein was bound to a nickel-charged affinity resin column, recombinant CsHSP17.2 (approximately 17 kDa) was separated from the fusion tag using the tobacco etch virus protease and then eluted from the column (Fig. 5A). To investigate whether CsHSP17.2 has molecular chaperone activity *in vitro*, citrate synthase (CS) were chemically denatured and renatured. When 300 nM CsHSP17.2 was added to the denatured CS under renaturing conditions, approximately 78% of the CS activity (threefold relative to the control) was recovered. In contrast, in the presence of H_2_O, only 26% of the CS activity was recovered after 75 min (Fig. 5B). These results suggested that CsHSP17.2 functioned as a molecular chaperone *in vitro*.

### Variations in thermotolerance, maximum photochemical efficiency (F_v_/F_m_), soluble protein and free proline content

**Figure 6:**
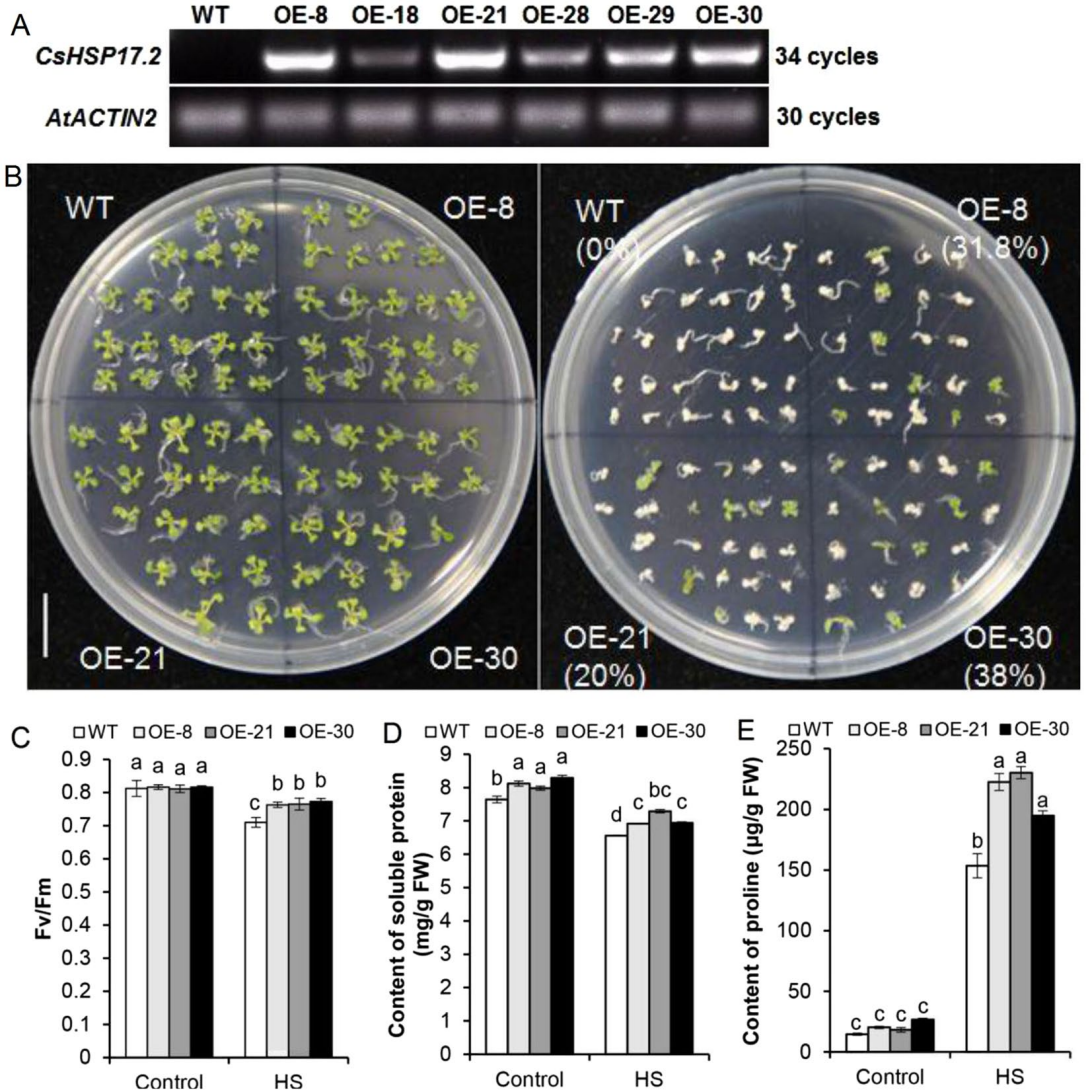
Thermotolerance of WT and transgenic *A. thaliana*. (**A**) RT-PCR identification of *CsHSP17.2* transgenic *A. thaliana* lines. Total RNA was extracted from rosette leaves of 4-week-old plants, and the *AtACTIN2* (AT3G18780) gene was used as a control for equal loading. (**B**) The survival rates of WT and transgenic *A. thaliana* plants under HS. The detection of chlorophyll fluorescence (**C**), soluble protein content (**D**) and proline content (**E**) in transgenic and WT plants. The *A. thaliana* seedlings were cultivated for four weeks under normal temperature (22 °C; Control) before HS (45 °C for 4 h), and the rosette leaves were harvested for the experiments. Data represent the means ± standard deviations of three replicates. Significant differences between WT and transgenic plants are indicated by different letters (*P* < 0.05). WT: wild-type; OE-8, OE-18, OE-21, OE-28, OE-29, and OE-30: six transgenic *A. thaliana* lines.

An RT-PCR analysis indicated that *CsHSP17.2* was substantially expressed in six independent transgenic *A. thaliana* lines (i.e., OE-8, OE-18, OE-21, OE-28, OE-29, and OE-30) but not in the wild-type (WT) plants (Fig. 6A). The three transgenic lines with the highest transgene expression levels (i.e., OE-8, OE-21, and OE-30) were used for subsequent experiments. The survival rates were higher in transgenic lines than in the WT plants after HS for 45 min, suggesting that *CsHSP17.2* overexpression enhanced thermotolerance in *A. thaliana* plants (Fig. 6B). To estimate the level of HS-induced damage to *A. thaliana* plants, related physiological indices were recorded after a 4-h HS treatment. Exposure to HS resulted in a significant decrease in the F_v_/F_m_ value in the *CsHSP17.2*-overexpressing and WT plants (*P* < 0.05) (Fig. 6C), but photosystem II was more active in the transgenic lines than in the WT plants, implying that *CsHSP17.2* overexpression stabilized F_v_/F_m_ under HS conditions. Similarly, the soluble protein content decreased more in WT plants than in the *CsHSP17.2-*overexpressing lines following HS treatment (Fig. 6D). This change indicated that *CsHSP17.2* overexpression resulted in increased amounts of soluble protein in transgenic plants. In addition, after exposure to HS, more free proline accumulated in the transgenic lines than in WT plants (Fig. 6E), indicating that *CsHSP17.2* overexpression promoted proline synthesis.

### *CsHSP17.2* overexpression in *A. thaliana* promotes seed germination and hypocotyl development under HS conditions

**Figure 7:**
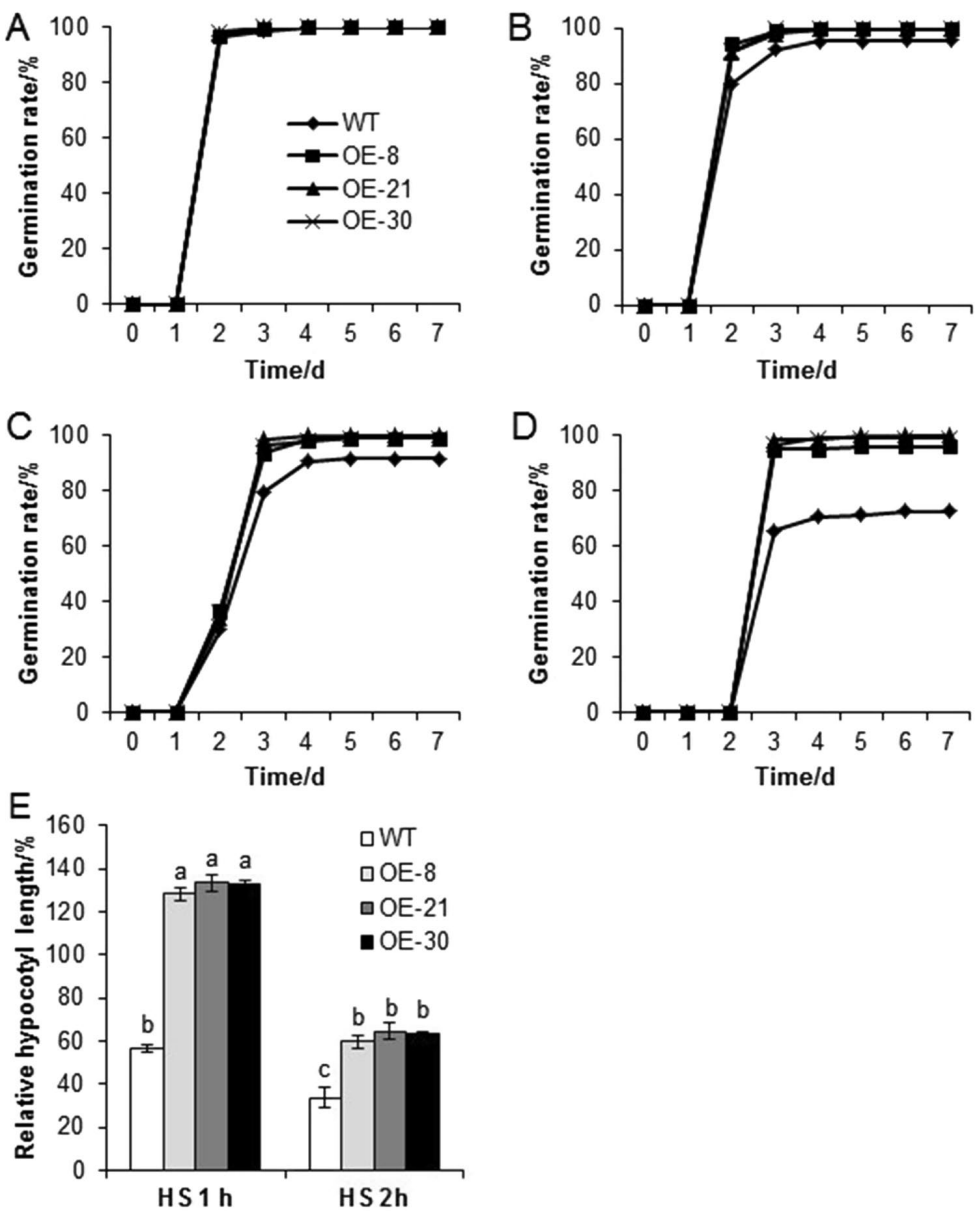
Germination rates (**A**–**D**) and relative hypocotyl lengths (**E**) of WT and transgenic *A. thaliana* under HS. (**A**) Control group, (**B**) HS for 1 h, (**C**) HS for 2 h, and (**D**) HS for 3 h. Error bars represent standard deviations and are based on data for at least 10 seedlings from each line. Significant differences between WT and transgenic plants are indicated by different letters (*P* < 0.05).

To determine whether CsHSP17.2 affected seed vigor, we examined the germination rates of WT and transgenic *A. thaliana* seeds under HS conditions. Without the HS treatment, nearly 100% of WT and transgenic seeds incubated at 22 °C germinated within 3 days (Figs 7A and S3a), indicating the high quality of the seeds used for the germination assays. However, the germination rates of transgenic and WT seeds gradually decreased with increasing duration of HS treatment. Additionally, the transgenic seeds exhibited enhanced vigor compared with the WT seeds after HS treatment (Figs 7B–D and S3b–d). Only 95.9% and 91.6% of WT seeds germinated after 1 and 2 h of heat treatment, respectively, whereas 98.7% of the transgenic seeds germinated even after 2 h. The germination rates of transgenic and WT seeds were 95.9–100.0% and 72.5% after a 3-h heat treatment, respectively. These results implied that *CsHSP17.2* overexpression in plants enhanced the basal thermotolerance of seeds.

Hypocotyls were much longer in the *CsHSP17.2-*overexpressing lines than in the WT plants after exposure to high temperatures for 1 or 2 h (Fig. 7E), indicating that *CsHSP17.2* plays an important role in hypocotyl development under HS conditions.

### Cloning of the putative promoter of *CsHSP17.2*

**Figure 8:**
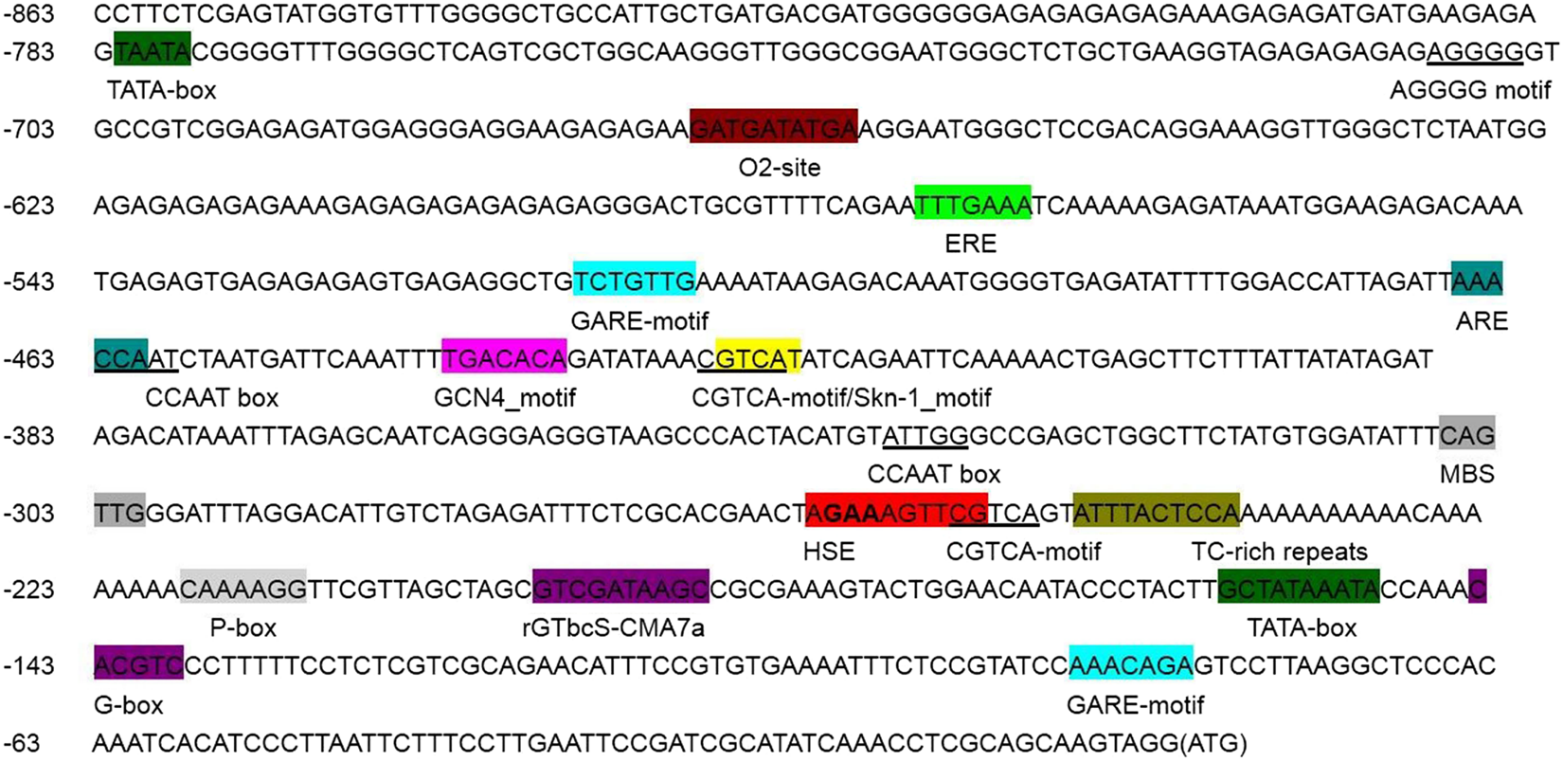
Analysis of the promoter sequences of *CsHSP17.2* using the PlantCARE promoter motif analysis tool. The start codon is shown in parentheses. The underlines and different colors signify different functional elements.

The 863-bp upstream sequence of the *CsHSP17.2* translation initiation site was obtained via thermal asymmetric interlaced PCR (TAIL-PCR) (Fig. S4), and the presence of core promoter elements was assessed using the PlantCARE database^29^. The presence of hormone-responsive motifs together with abiotic stress-related motifs and developmental stage specific elements was observed in the putative promoter sequence (Fig. 8 and Table S1). The presence of these regulatory elements suggested that the promoter region of *CsHSP17.2* might be responsive to a wide variety of abiotic stress and hormones and induced in some developmental stages.

### Responsiveness of the *CsHSP17.2* promoter to abiotic stress and hormones

**Figure 9:**
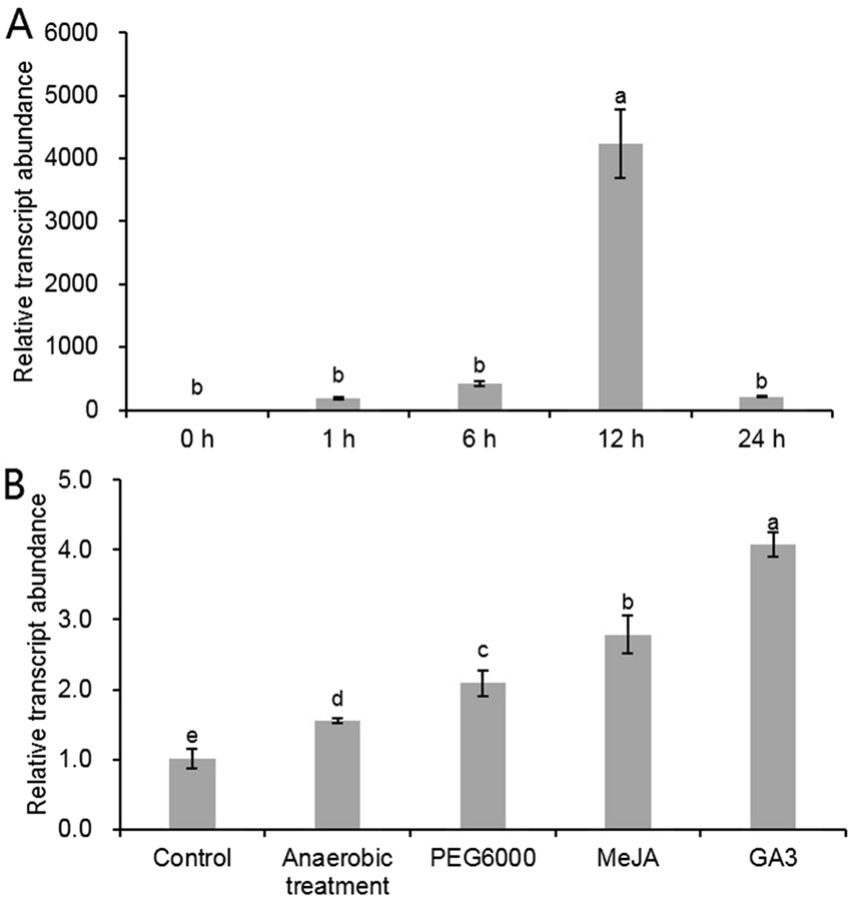
Induction of *GUS* gene governed by the *CsHSP17.2* promoter. (**A**) qRT-PCR for the *GUS* transcript under heat stress. (**B**) qRT-PCR for the *GUS* transcript under other abiotic stress and hormone treatments. Data represent the means ± standard deviations of four replicates, and the significant differences are indicated by different letters (*P* < 0.05).

The induction of the GUS under abiotic stress and hormones was monitored in 3-week-old transgenic *A. thaliana* plants via quantitative real-time PCR (qRT-PCR) and GUS histochemical staining. The *GUS* transcript gradually increased and peaked at approximately 12 h of heat stress treatment; thereafter, the transcription levels decreased significantly (Fig. 9A). The GUS histochemical assay revealed protein accumulation until 24 h, suggesting that although the transcript might not be required in high amounts, the corresponding protein accumulated (Fig. S5).

In addition to heat stress, the *CsHSP17.2* promoter activity was also induced by simulated drought (10% PEG6000) and plant hormones (40 μM MeJA or 20 μM GA3) (Figs 9B and S5). These findings were consistent with the presence of multiple abiotic stress- and hormone-responsive elements in the *CsHSP17.2* promoter sequence.

## Discussion

Since the first sHSP was discovered in *Drosophila melanogaster*, numerous sHSPs have been identified in various plant species^5, 30^. Plant sHSPs are classified into different subfamilies based on amino acid sequence similarities and localization to distinct subcellular compartments. In this study, the green fluorescence of the CsHSP17.2:GFP fusion protein was detected in the cytosol and nucleus, in agreement with our results from analyses of homology and phylogenetic relationships. These findings suggested that CsHSP17.2 is a Class I sHSP.

Numerous studies have previously demonstrated that *sHSPs* are regulated by various abiotic stress. A *Primula* sHSP gene, *PfHSP17.1*, is induced by heat, cold, salt, PEG-induced drought stress, and oxidative stress^14^. Similarly, a *Tamarix hispida* sHSP gene, *ThHSP18.3*, is induced by cold, heat, salt, and drought stress^12^. In addition, a *David Lily* sHSP gene, *LimHSP16.45* is triggered by cold and heat stress^16^. Our observations revealed that the expression of *CsHSP17.2* was differentially regulated by heat, cold, drought, salinity, and ABA treatments, suggesting that it has different roles in various abiotic stress responses. However, *CsHSP17.2* expression was not obviously affected by cold stress, which suggested that *CsHSP17.2* might not participate in the cold stress response. Interestingly, *CsHSP17.2* transcription was down-regulated by ABA treatment, which was consistent with the results of Sun *et al*.^31^, suggesting that *CsHSP17.2* may function in an ABA-dependent manner.

Several recent studies have used the expression of *sHSP* genes in *E. coli* cells to investigate their possible functions *in vivo*. The heterologous expression of a *Medicago sativa* mitochondrial *HSP23* protected *E. coli* from salinity and arsenic stresses^32^, and the overexpression of a *Rosa chinensis RcHSP17.8* enhanced *E. coli* viability during exposure to heat and cold stresses^33^. Additionally, as a major eukaryotic model organism, *P. pastoris* produces sHSPs and is often used to investigate sHSP functions^33, 34^. Similarly, the *E. coli* and *P. pastoris* cells constitutively expressing *CsHSP17.2* were more viable when exposed to heat stress than the control cells, implying that *CsHSP17.2* overexpression results in greater thermotolerance.

The F_v_/F_m_ value is a good indicator of the photosynthetic functions of plants under adverse environmental conditions. A previous study concluded that overexpression of a tomato (*Lycopersicon esculentum*) *HSP21* protected photosystem (PS) II from the effects of temperature-dependent oxidative stress^35^. In our study, the F_v_/F_m_ values of *CsHSP17.2*-overexpressing plants were significantly higher than those of WT plants under HS conditions, suggesting that CsHSP17.2 has a role in protecting of PSII against oxidative stress created by high temperature. Previous studies have demonstrated that protein synthesis positively correlates with stress tolerance, and heat-tolerant plants maintain a higher protein synthesis rate and a lower protein degradation rate than heat-intolerant plants^36, 37^. The overall protein levels in *A. thaliana* expressing exogenous *CsHSP17.2* significantly increased compared with WT plants, indicating that *CsHSP17.2* overexpression enhances thermotolerance by facilitating protein synthesis. It has been well established that excess H_2_O_2_ in plant cells leads to the occurrence of oxidative stress^38, 39^. In our study, the histochemical detection of H_2_O_2_ indicated that *CsHSP17.2* overexpression resulted in increased scavenging of H_2_O_2_ under HS conditions (Fig. S6), suggesting that *CsHSP17.2* overexpression confers thermo-tolerance in *A. thaliana*. Ascorbate peroxidases (APXs) and peroxidases (PODs) are considered to have essential roles in scavenging reactive oxygen species (ROS) and protecting cells from oxidative damage in higher plants^40, 41^. During exposure to HS, the transcription levels of *AtAPX1* and *AtPOD* were higher in the *CsHSP17.2*-overexpressing lines than in the WT plants (*P* < 0.05) (Fig. S7). Consequently, we hypothesize that *CsHSP17.2* increases the capacity for ROS-scavenging by elevating *AtAPX1* and *AtPOD* transcript levels. Proline confers osmotic tolerance during exposure to stress conditions, and *AtP5CS2* and *AtProT1* are two key genes involved in proline biosynthesis and transport^42, 43^. The transcription levels of these two genes were higher in the transgenic lines than in WT plants under normal and HS conditions (Fig. S7), which was consistent with the content of proline, implying that *CsHSP17.2* overexpression may enhance proline biosynthesis and transport and ultimately increase the thermotolerance in *A. thaliana*.

Heat shock transcription factors (HSFs) are transcription factors that activate the expression of genes in response to stress, thereby playing a central role in cellular homeostatic control mechanisms^44^. An HSF binds to a DNA sequence motif, the heat shock element (HSE), which is characterized by an array of inverted repeats of the motif nGAAn. Besides an HSE motif, AGGGG motif (designated as STRE) and CCAAT box were also found in the promoter region of *CsHSP17.2*, which have been demonstrated to act cooperatively with the HSEs to enhance HSP promoter activity under high temperature^45, 46^. Therefore, these combined results strongly suggest that *CsHSP17.2* is regulated by HSFs and induced by heat stress conditions. The transcript levels of *AtHSF* genes (especially *AtHSFA4a* and *AtHSFC1*) were higher in *CsHSP17.2* transgenic lines than in WT plants. Thus, we speculated that *CsHSP17.2* overexpression might enhance the thermotolerance of transgenic plants by co-regulating *HSF* gene expression.

Molecular chaperones, including sHSPs, are important for maintaining cellular homeostasis under optimal and adverse growth conditions. They can bind to partially denatured proteins to prevent further denaturation or aggregation and promote the correct refolding of proteins^4, 47^. Our results indicated that CsHSP17.2 functions as a molecular chaperone *in vitro* by inducing the renaturation and reactivation of chemically denatured CS, suggesting that CsHSP17.2 may help to maintain proteins in their functional conformations and to prevent the aggregation of non-native proteins in host cells. *AtHSP17.4* and *AtHSP70*, which are associated with molecular chaperone activities^11, 48, 49^, were more actively transcribed in transgenic *A. thaliana* plants under HS conditions (Fig. S7). These findings suggested that *CsHSP17.2* overexpression induces HSP synthesis and confers thermotolerance in plants.

The sHSPs have protective roles in seed germination. Transgenic *A. thaliana* seeds expressing *NnHSP17.5* displayed enhanced seed germination vigor to heat stress^50^. Similarly, ectopic expression of *LimHSP16.45* enhanced the seed viability of *A. thaliana* exposed to high temperature, salinity, and oxidative stress^51^. In this study, seeds from *CsHSP17.2*-overexpressing lines exhibited enhanced germination rates under HS conditions, suggesting that *CsHSP17.2* may contribute to basal thermotolerance during seed germination. Induced thermotolerance is defined as the capacity of an organism to survive a normally lethal temperature if it is first conditioned by pretreatment at a milder temperature and can be measured in hypocotyl elongation assays^52^. *CsHSP17.2*-overexpressing lines exhibited higher hypocotyl elongation lengths, suggesting that *CsHSP17.2* overexpression conferred induced thermotolerance in transgenic plants. To the best of our knowledge, this report is the first demonstrating that a Class I sHSP is correlated with induced thermotolerance. Thus, we speculate that CsHSP17.2 is essential for basal and induced thermotolerance in transgenic *A. thaliana*.

Previous studies have demonstrated that overexpression of *sHSPs* have different roles in plant growth and development. Overexpression of *TaHSP26* in *A. thaliana* produced higher biomass and seed yield than WT plants^3^. Besides, transgenic tomato (*Lycopersicon esculentum*) plants constitutively expressing *LeHSP21* promoted fruit ripening under normal growth temperature^35^. However, overexpression of *NnHSP17.5* had no positive or detrimental effects on plant development in *A. thaliana*. Similarly, overexpressing *CsHSP17.2* did not cause any obvious phenotypic changes. The germination times and rates, growth rates, time to flowering, and seed yields of the transgenic plants were similar to those of WT plants (Fig. S8). For example, under normal conditions, there were no significant differences between WT and *CsHSP17.2*-overexpressing plants in terms of the fresh weights of rosette leaves from 4- or 5-week-old seedlings (Fig. S9). These results suggested that heterologous expression of *CsHSP17.2* may be used for enhancing thermotolerance in economically important crops.

In summary, our results demonstrate that *CsHSP17.2* is a heat-inducible gene, and CsHSP17.2 has molecular chaperone activities *in vitro*. *CsHSP17.2* has the capacity to confer thermotolerance not only on *E. coli* and *P. pastoris*, but also on *A. thaliana* under heat stress. We propose that *CsHSP17.2* increases plant thermotolerance through several pathways, including the maintenance of photosynthetic rates and protein synthesis, enhancement of ROS-scavenging, and expression of HS-responsive genes. These findings should help to clarify the complex mechanisms and roles of HSPs in regulating plant responses to environmental stresses.

## Methods

### Plant materials and stress treatments

Two-year-old *C. sinensis* cv. ‘Yingshuang’ plants were grown in a light incubator with a 12-h light (200 μmol·m^−2^·s^−1^; 24 °C)/12-h dark (20 °C) photoperiod for 30 days before treatments. For HS treatments, plants were placed in a light incubator (40 °C) for 24 h. For recovery treatments, the plants (HS for 1 h) were allowed to recover at 24 °C for 3 h, and samples were collected every hour. For the low-temperature treatment, tea plants were transferred to another chamber maintained at 4 °C. To simulate high salinity and drought stresses, tea plants were treated with 300 mM NaCl and 20% (w/v) PEG6000, respectively. For the exogenous ABA treatment, tea leaves were sprayed with 50 µM ABA solution. All treatments were completed under normal conditions (as described above), unless otherwise indicated. Additionally, the third fully expanded leaves from the top buds were harvested at 0, 1, 6, 12, and 24 h after various treatments, immediately frozen with liquid nitrogen, and stored at −70 °C for subsequent RNA extraction.

### Molecular cloning of *CsHSP17.2* gene and its promoter

Total RNA was isolated from tea leaves using a Quick RNA Isolation Kit (Huayueyang, Beijing, China), and 1 μg of total RNA was reverse transcribed to generate first-strand cDNA using Reverse Transcriptase M-MLV (RNase H−) (TaKaRa, Dalian, China) according to the manufacturer’s instructions. To obtain the ORF of *CsHSP17.2*, a primer pair (ORF-F/-R, Table S2) was designed and employed for PCR amplification. The resulting amplicons were purified and cloned into the pEASY-T1 Simple Cloning Vector (Transgen, Beijing, China) for sequencing (Genscript, Nanjing, China).

Genomic DNA was extracted from tea buds using Plant Genomic DNA Kit (TIANGEN, Beijing, China) according to the manufacturer’s instructions. The promoter of *CsHSP17.2* gene was amplified using Genome Walking Kit (TaKaRa, Dalian, China) with three gene-specific primers (SP1, SP2 and SP3, Table S2). The target products were cloned into the pEASY-T1 simple vector and sequenced. A search for regulatory elements in the promoter was performed using the PlantCARE (Plant Cis-Acting Regulatory Element) database^29^.

### Analyses of sequences and phylogenetic relationships

The ClustalX program was used for multiple sequence alignments^53^, and a phylogenetic tree was constructed using MEGA5 according to the neighbor-joining method^54^.

### Subcellular localization of the CsHSP17.2 protein in onion (*Allium cepa*) epidermal cells and tobacco (*Nicotiana benthamiana*) leaves

To construct a transient expression vector, the ORF of the *CsHSP17.2* gene (without the stop codon) was amplified using the sub-F/-R primer pair (Table S2). The amplified *CsHSP17.2* coding region was inserted into the pCAMBIA2300-GFP vector at the *Bam* HI and *Xba* І sites to generate the *CsHSP17.2*::*GFP* construct. Onion epidermal cells were transformed with the recombinant plasmid or the empty vector using a PDS-1000/He particle delivery system (Bio-Rad, Hercules, CA, USA). The transformed onion epidermal cells were then incubated on Murashige and Skoog (MS) agar medium for 16 h at 25 °C in darkness. Finally, the GFP signal was detected using a Zeiss LSM700 confocal laser-scanning microscope (Carl Zeiss Inc., USA).

For transient expression in tobacco cells, *Agrobacterium tumefaciens* strain GV3101 cells were independently transformed with the recombinant plasmid or empty vector. The transformed *A. tumefaciens* cells were infiltrated into tobacco leaves^55^, and then plants were incubated for 3 days in darkness. The GFP signal was detected as described above.

### Gene expression analysis by qRT-PCR

Total RNAs were extracted from tea leaves exposed to different treatments using the Quick RNA Isolation Kit (Huayueyang, Beijing, China). After assessing the quality and determining the concentration of total RNA using the ONE Drop OD-1000+ spectrophotometer (ONE Drop, USA), 1 μg of total RNA was reverse transcribed to single-stranded cDNA using the PrimeScript RT Reagent Kit with gDNA Eraser (TaKaRa, Dalian, China) following the manufacturer’s instructions. The CsHSP17.2-qF/-qR primer pair (Table S2) was used to analyze *CsHSP17.2* expression levels. The *C. sinensis β-actin* gene (GenBank accession number: HQ420251, Table S2) was used as the reference gene.

*A. thaliana* transgenic plants (3-week-old) were exposed to various abiotic stress and hormone treatments. For heat stress treatment, plants were cultured in a growth cabinet at 40 °C (dark) for different time periods. Drought stress was provided by treating with 10% PEG6000 for 4 h, and anaerobic treatment by immersing seedlings in distilled water for 3 h. For hormone treatments, leaves of plants were sprayed with 40 μM methyl jasmonate (MeJA) or 20 μM gibberellin A3 (GA3) for 24 h. After these treatments, the *A. thaliana* rosette leaves were sampled and flash-frozen in liquid nitrogen and stored at −80 °C for RNA extraction. All of these treatments were performed under a growth regime of 16/8 h light/darkness at 22 °C unless otherwise mentioned. qRT-PCR was performed with *GUS*-specific primers (GUS-qF/-qR, Table S2), and *AtACTIN2* (AT3G18780) was selected as a housekeeping gene.

To analyze the expression profiles of HS-responsive genes, total RNA was extracted from the leaves of 4-week-old *A. thaliana* plants that had been incubated at 45 °C for 0, 0.5, 1, 2, and 4 h. The expression profiles of twelve HS-responsive genes were determined by qRT-PCR, with the *A. thaliana ACTIN2* (AT3G18780) gene used as the housekeeping gene. All of the relevant primers are listed in Table S3.

The qRT-PCR was completed using SYBR Premix Ex Taq II (Tli RnaseH Plus) (TaKaRa, Dalian, China) and an iQ5 Multicolor Real-Time PCR Detection System (Bio-Rad). Each 20 μl qRT-PCR sample consisted of 10 μl of SYBR Premix Ex Taq II (2x), 0.2 μM of each primer, and 10 ng of cDNA template. The PCR program was as follows: 95 °C for 30 s, 40 cycles at 95 °C for 5 s, and 58 °C for 30 s. All experiments were repeated three times with independent RNA samples, and the relative expression levels were calculated using the 2^−ΔΔ*C*T^ method^56^.

### *CsHSP17.2* overexpression in *E. coli* cells

The full-length *CsHSP17.2* coding region was amplified using the pro-F/-R primer pair (Table S2) and KOD FX Neo polymerase (TOYOBO, Shanghai, China). The amplicons were subcloned into the pEASY-Blunt Simple Cloning Vector (Transgen, Beijing, China). The intermediate vector was digested with *Bam* HІ and *Eco* RІ (Thermo Scientific, Waltham, USA), and the *CsHSP17.2* fragment was ligated into a pET-32a(+) vector digested with the same enzymes. The pET-CsHSP17.2 recombinant plasmid and the empty vector were used to transform *E. coli* BL21 (DE3) cells. The fusion proteins were analyzed via sodium dodecyl sulfate polyacrylamide gel electrophoresis (SDS-PAGE) according to the previously described methods and techniques^57^.

### Thermotolerance of transgenic *E. coli* exposed to HS

Thermotolerance assays were conducted according to the method described by Soto *et al*.^58^ with some modifications. Briefly, when the cultures (grown at 37 °C) of *E. coli* harboring the pET-CsHSP17.2 plasmid or the empty vector reached an optical density (at 600 nm, OD_600_) of 1.0, they were diluted 100-fold with fresh Luria-Bertani liquid medium supplemented with ampicillin (100 μg·ml^−1^ final concentration). After the *E. coli* cells were treated with 1 mM IPTG for 4 h, 1-ml samples were transferred to a temperature-controlled water bath (50 °C), and 30-μl aliquots were added to Luria-Bertani agar plates after 0, 30, 60, and 90 min. Cell viability was estimated by counting the number of colony-forming units after an overnight incubation at 37 °C.

### CsHSP17.2 purification and chemical denaturation and renaturation experiments

Recombinant proteins were purified using a nickel-charged affinity resin column (Qiagen GmbH, Germany) according to the method described by Liu *et al*.^8^. The fusion proteins were cleaved with tobacco etch virus protease for 4 h at 30 °C and then analyzed by SDS-PAGE. Gels were stained with Coomassie brilliant blue R-250 and photographed. The concentrations of the purified proteins were determined using the Bradford method^59^ with bovine serum albumin (BSA) as the standard.

Refolding assays involving chemically denatured citrate synthase (CS) were conducted as described by Collada *et al*.^60^ to determine whether CsHSP17.2 exhibited molecular chaperone activities. First, 15 μM CS (Sigma, St. Louis, MO, USA) was denatured with 6 M guanidine hydrochloride for 2 h, and then diluted 100-fold in refolding buffer (100 mM Tris-HCl, pH 8.0) supplemented with 300 nM CsHSP17.2 protein or distilled water. We then analyzed 20-μl aliquots for CS activity by monitoring the breakage of the thioester bond of acetyl CoA, which absorbs at 233 nm.

### Constitutive expression of *CsHSP17.2* in *P. pastoris*

The coding region of *CsHSP17.2* was inserted into the pPIC3.5 K vector (Invitrogen, Carlsbad, CA, USA) to construct a *P. pastoris* overexpression plasmid. After being linearized with *Sal* I (Thermo Scientific, Waltham, USA), the recombinant vector (pPIC3.5K-HSP17.2) and the empty vector pPIC3.5 K were independently inserted into *P. pastoris* strain SMD1168 cells using the MicroPulser electroporator (Bio-Rad). Positive clones were identified by PCR with the ORF-F/3′AOX1 primer pair and the universal primers 5′AOX1/3′AOX1 (Table S2).

For thermotolerance assays, cultures of *P. pastoris* carrying pPIC3.5K-HSP17.2 (OD_600_ = 1.5) were incubated at 50 °C for 0, 30, and 60 min, and 10-μl aliquots of 10-fold serial dilutions were spotted onto yeast extract/peptone/dextrose (YEPD) agar medium. After incubation at 30 °C for 3 days, samples were observed and photographed using a digital camera (Canon, Japan). The *P. pastoris* strains harboring the pPIC3.5 K were used as controls.

### Plasmid construction and transformation of *A. thaliana*

To investigate the functions of CsHSP17.2, a plant expression vector containing *35S*:*CsHSP17.2*:*GFP* (Fig. S10a) was constructed and inserted into *A. tumefaciens* strain EHA105 cells. A floral dip method^61^ was used to transform *A. thaliana*. Transgenic *A. thaliana* plants were screened on 1/2 MS agar medium supplemented with 50 μg·ml^−1^ kanamycin. The T_3_ generations were analyzed by RT-PCR with gene-specific primers (ORF-F/-R, Table S2) before being used in subsequent experiments. All plants were grown at 22 °C in a light incubator with a 16-h light (200 μmol·m^−2^·s^−1^)/8-h dark cycle unless otherwise specified.

For characterization, the putative *CsHSP17.2* promoter (*PHSP17.2*) was cloned into the pEASY-T1 Simple Cloning Vector with the primer pair pHSP17.2-F/-R (Table S2). Then, the amplified product was digested with *Hin*d III and *Bam* HI and inserted into the pBI121 vector (Invitrogen) to generate the *PHSP17.2*:*GUS* fusion vector (Fig. S10b). *Agrobacterium* strain EHA105 harboring the binary vector *PHSP17.2*:*GUS* was used for *A. thaliana* transformation. The independent transformants were selected on 1/2 MS agar medium containing 50 μg·ml^−1^ kanamycin and the T_3_ homozygous plants were raised for subsequent histochemical staining and *GUS* transcription assays. The PCR primers used to confirm the transgenic *A. thaliana* were T-pHSP17.2-F and T-pHSP17.2-R (Table S2).

### Thermotolerance test

Plates containing 1-week-old *A. thaliana* plants were submerged in a water bath at 45 °C for 45 min according to the method described by Charng *et al*.^62^.

### Chlorophyll fluorescence, soluble protein and proline content measurements and H_2_O_2_ detection

The chlorophyll fluorescence of rosette leaves was measured using an Imaging-PAM Chlorophyll Fluorometer (M-series; Heinz Walz GmbH, Germany). All *A. thaliana* plants were incubated in darkness for 10 min immediately before measurements, and the F_v_/F_m_ was calculated automatically. The soluble protein contents were analyzed using Coomassie brilliant blue G250 according to a published procedure^37^. The free proline content was determined using the acid-ninhydrin method^63^. We detected H_2_O_2_ in *A. thaliana* rosette leaves according to the method described by Orozco-Cardenas and Ryan^64^. For these experiments, 4-week-old seedlings were incubated at 45 °C for 4 h, and each experiment was performed three times.

### Germination assays

Seeds of WT and *CsHSP17.2-*overexpressing plants (i.e., OE-8, OE-21, and OE-30) were plated on 1/2 MS medium. After a 3-day cold treatment (4 °C) in darkness, the plates were transferred to a water bath at 45 °C and incubated for 0, 1, 2, and 3 h. The plates were then placed in a light incubator under normal conditions for 7 days, and the germination rates were calculated every day. After the 7-day incubation, representative plates were photographed.

### Hypocotyl elongation assays

Hypocotyl elongation assays were conducted according to a published method^52^ with minor modifications. For all assays, seeds were plated on 1/2 MS medium, and the plates were covered with foil. After a 2.5-day cold treatment, the plates were incubated at 22 °C for an additional 2.5 days. The seeds were then incubated at 38 °C for 90 min, followed by a 2-h recovery period at 22 °C and then a 1- or 2-h treatment at 45 °C (i.e., HS treatment). After another 2.5 days, the hypocotyl lengths of all seedlings were measured. The relative hypocotyl length was calculated using the following formula: (hypocotyl lengths of 5-day-old seedlings under HS conditions–hypocotyl lengths of 2.5-day-old seedlings)/hypocotyl lengths of 2.5-day-old seedlings. Experiments included at least 10 seedlings from each line and were repeated at least three times.

### Phenotypic analysis

The phenotypes of each *A. thaliana* line were observed and photographed after 7 days (grown in 1/2 MS), 14 days (grown in 1/2 MS), 5 weeks (grown in soil), and 7 weeks (grown in soil), respectively. Additionally, the fresh weights of rosette leaves were measured after 4 and 5 weeks of cultivation under normal conditions.

### Histochemical GUS staining

The GUS histochemical staining of transgenic *A. thaliana* plants containing the *PHSP17*.*2*:*GUS* fusion construct followed the method described previously^65^. The explants were then observed with a bright field microscope and photographed (Leica Q500MC, Cambridge, England).

### Statistical analysis

All data were statistically analyzed with SPSS 17.0 software (SPSS Inc., Chicago, IL, USA) using Duncan’s multiple range test at a 0.05 level of significance.

## Electronic supplementary material

Supplementary Information
