# Supplementary material for: The CsHSP17.2 molecular chaperone is essential for thermotolerance in Camellia sinensis
Source: Sci Rep. 2017 Apr 27;7:1237. doi: 10.1038/s41598-017-01407-x (PMC5430664; doi:10.1038/s41598-017-01407-x)
Supplement: Supplementary file 1 — Supplementary Information [file 41598_2017_1407_MOESM1_ESM.pdf]

The CsHSP17.2 molecular chaperone is essential for thermotolerance in *Camellia sinensis*

Mingle Wang[1], Zhongwei Zou[2], Qinghui Li[1], Kang Sun[1], Xuan Chen[1] & Xinghui Li[1,*]

[1]Tea Research Institute, Nanjing Agricultural University, Nanjing, Jiangsu Province, P. R. China

[2]Department of Plant Science, University of Manitoba, Winnipeg, Manitoba, Canada

* Correspondence: Dr. Xinghui Li

Tea Research Institute, Nanjing Agricultural University, Weigang No. 1, Nanjing, Jiangsu Province, P. R. China

Tel: +86 25 8439 5182

Fax: +86 25 8439 5182

E-mail: lxh@njau.edu.cn

Supplementary Information

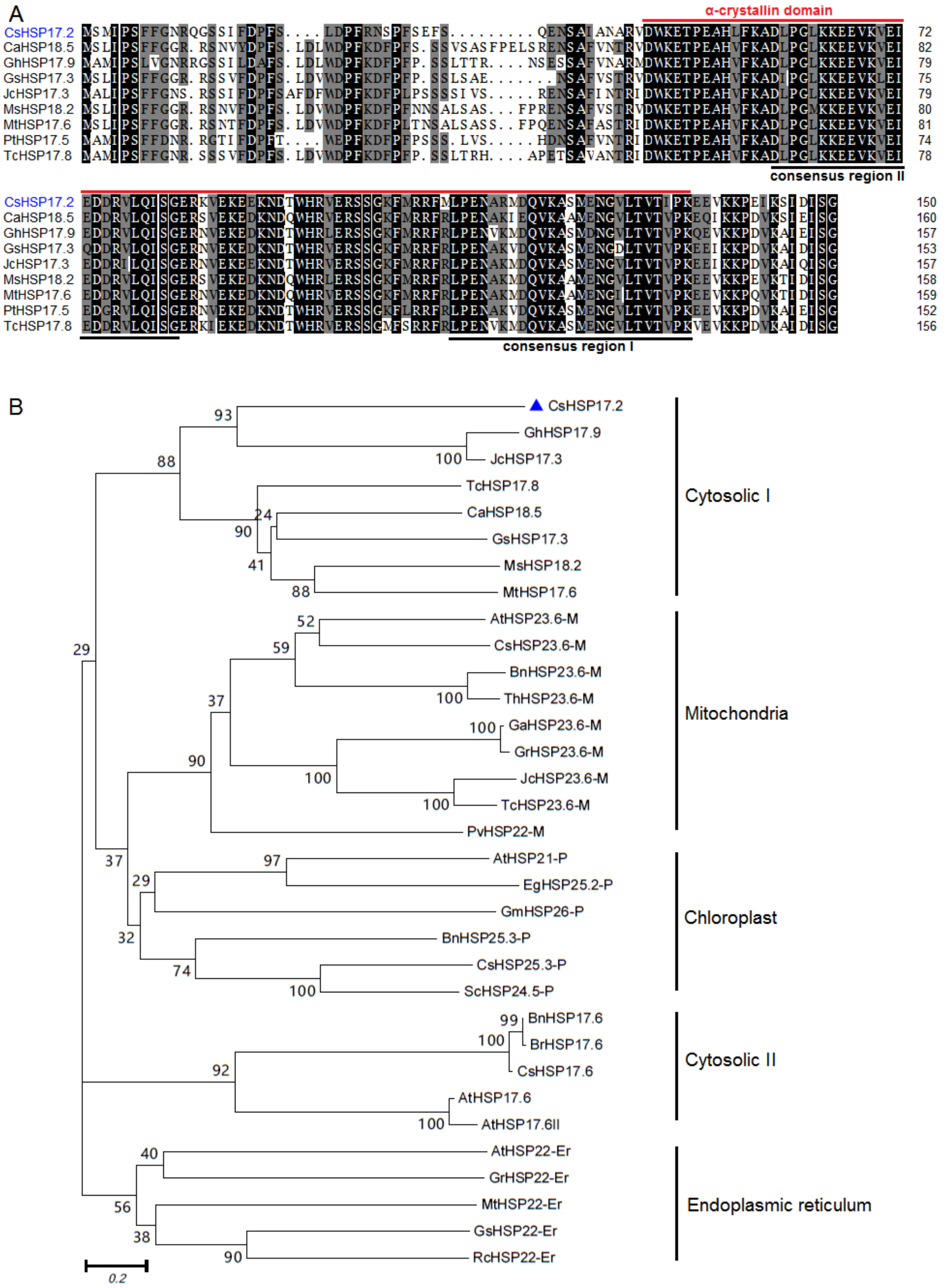

Figure S1. Characterization of CsHSP17.2 protein. (**a**) Comparison of the deduced protein sequences of CsHSP17.2 with other plant sHSPs. The α-crystallin domain and two consensus regions are highlighted by *red* and *black* lines, respectively. (**b**) Phylogenetic analysis of the relationship between CsHSP17.2 and sHSPs from other plant species. The Neighbor-Joining

method was used with the following parameters: bootstrap (1 000 replicates), poisson correction and complete deletion. The genetic distances are indicated by the horizontal bar. The amino acid sequences are shown as follows: AtHSP21-P (AAA32818), AtHSP17.6 (NP_196763), AtHSP17.6II (BAC43441), AtHSP22-Er (NP_192763), and AtHSP23.6-M (NP_194250), *Arabidopsis thaliana*; BnHSP25.3-P (XP_013698943), BnHSP17.6 (XP_013712624), and BnHSP23.6-M (XP_013740237), *Brassica napus*; BrHSP17.6 (XP_009125845), *Brassica rapa*; CaHSP18.5 (XP_004505085) and CaHSP22.7-Er (XP_004495437), *Cicer arietinum*; CsHSP25.3-P (XP_010438568), CsHSP17.6 (XP_010453267), and CsHSP23.6-M (XP_010448463), *Camelina sativa*; CsHSP17.2 (KU244518), *Camellia sinensis*; EgHSP25.2-P (XP_010035308), *Eucalyptus grandis*; GaHSP23.6-M (KHG21474), *Gossypium arboreum*; GhHSP17.9 (AEH30706), *Gossypium hirsutum*; GmHSP26-P (XP_003523325), *Glycine max*; GrHSP22-Er (XP_012462416) and GrHSP23.6-M (XP_012439144), *Gossypium raimondii*; GsHSP17.3 (KHN46262) and GsHSP22-Er (KHN30529), *Glycine soja*; JcHSP17.3 (XP_012066921) and JcHSP23.6-M (XP_012090444), *Jatropha curcas*; MsHSP18.2 (P27880), *Medicago sativa*; MtHSP17.6 (XP_003619703) and MtHSP22-Er (XP_013468797), *Medicago truncatula*; PtHSP17.5 (XP_002325979), *Populus trichocarpa*; PvHSP22-M (AHA84250), *Phaseolus vulgaris*; RcHSP22-Er (XP_015575066), *Ricinus communis*; ScHSP24.5-P (ABD66589), *Senecio crataegifolius*; TcHSP17.8 (XP_007016257) and TcHSP23.6-M (XP_007038384), *Theobroma cacao*; ThHSP23.6-M (XP_010532236), *Tarenaya hassleriana*.

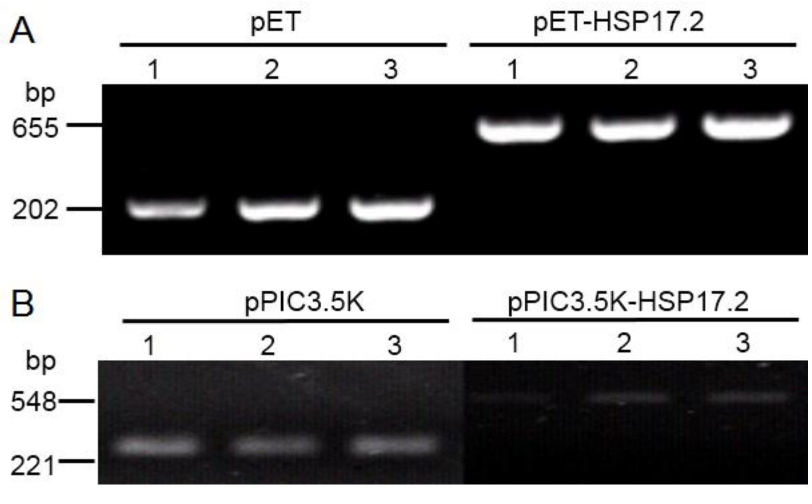

Figure S2. Confirmation of the positive clones in (**a**) ***E. coli*** **and** (**b**) ***P. pastoris*****.** pET and pPIC3.5K indicate two different empty vectors in *E. coli* and *P. pastoris*, respectively, while

pET-HSP17.2 and pPIC3.5K-HSP17.2 are the corresponding recombinant plasmids. *1*, *2*, and *3*: three independent transformants.

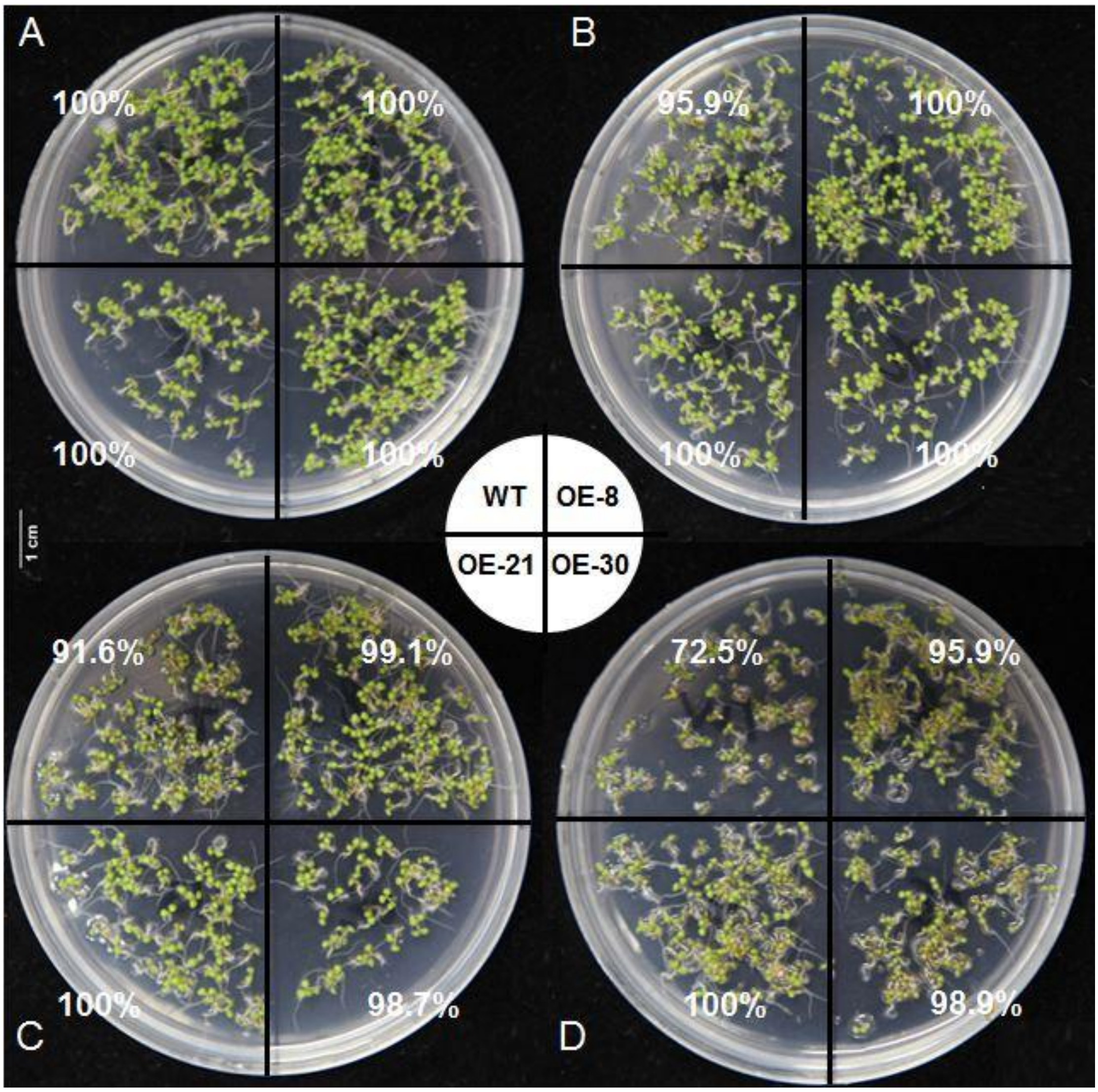

Figure S3. The germination performance of *A. thaliana* seeds under HS (45 ℃). (**a**) The control group untreated with HS. (**b**) HS for 1 h. (**c**) HS for 2 h. (**d**) HS for 3 h. WT: wild-type. OE-8, OE-21, and OE-30: transgenic *A. thaliana* lines. *Scale bar* = 1 cm.

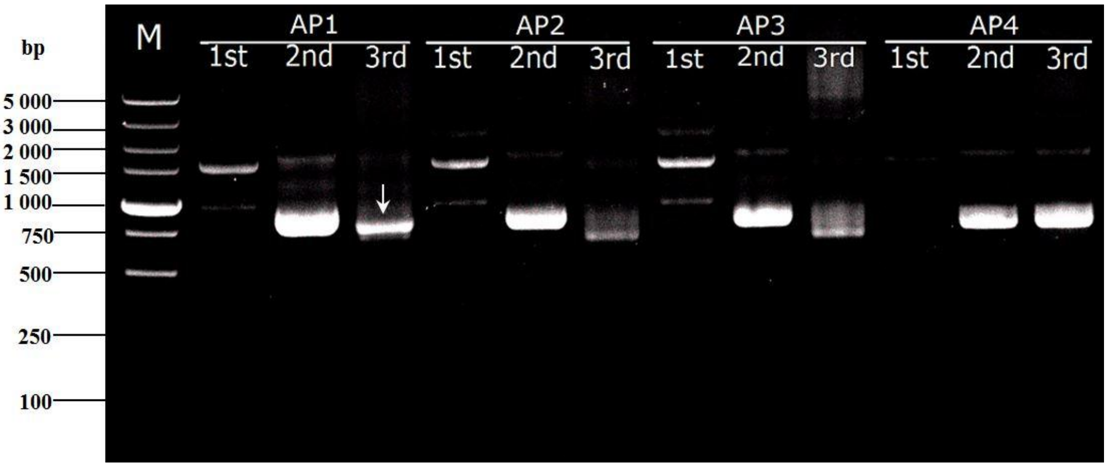

Figure S4. Isolation of *CsHSP17.2* promoter by thermal asymmetric interlaced PCR (TAIL-PCR). *M*, DL5000 DNA marker. *AP1*, *AP2*, *AP3*, and *AP4*: the degenerate primers provided by Genome Walking Kit (TaKaRa, Dalian, China). *1st*, *2nd*, and *3rd*: the PCR products of the primary, secondary, and tertiary reactions, respectively. The target fragment was indicated by *white arrow*.

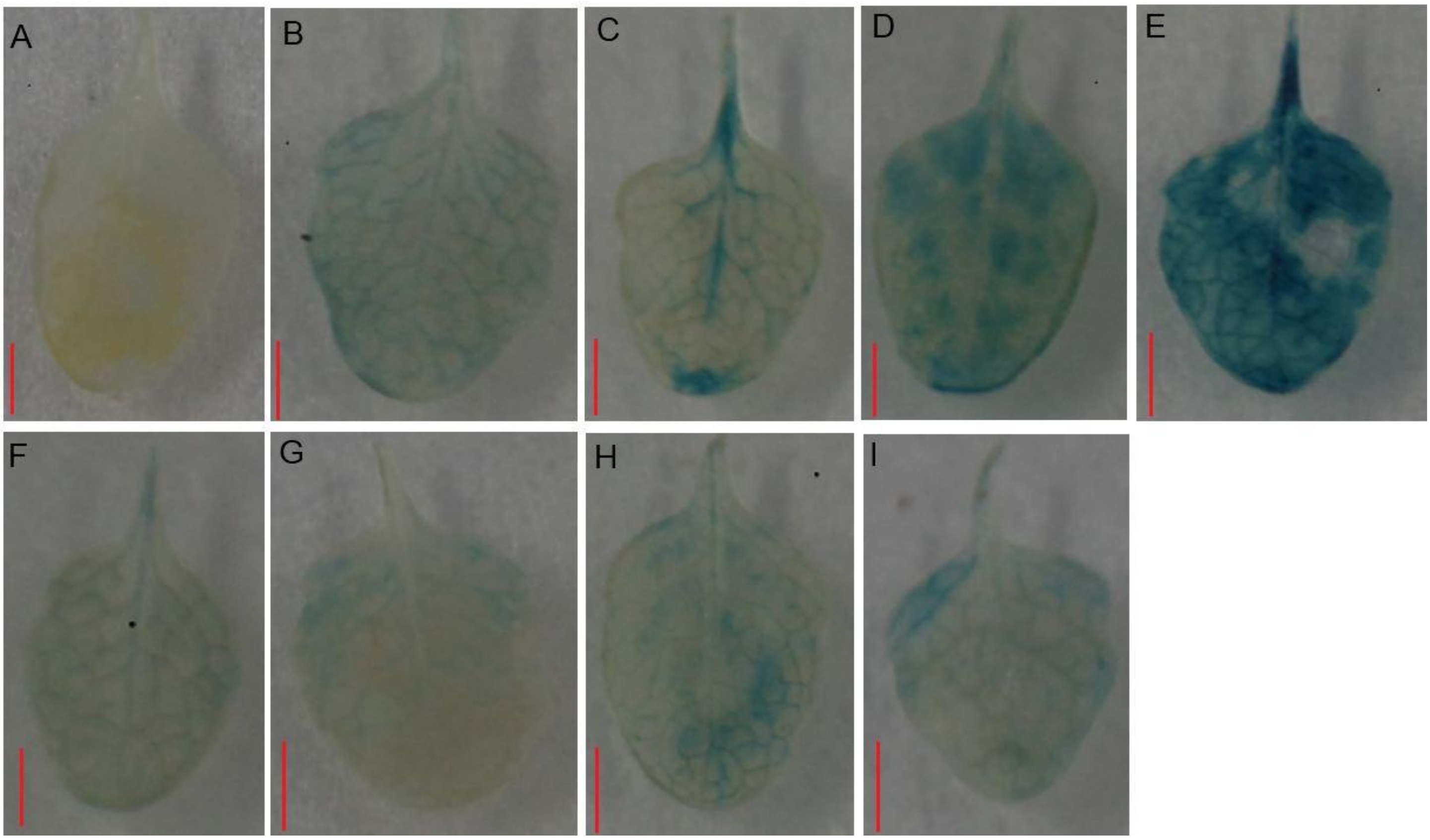

Figure S5. GUS histochemical assay under abiotic stresses and hormones. GUS accumulation under heat stress for (**a**) 0 h, (**b**) 1 h, (**c**) 6 h, (**d**) 12 h, and (**e**) 24 h. GUS accumulation under (**f**) anaerobic treatment, (**g**) PEG6000, (**h**) MeJA, and (**i**) GA3. *Scale bar* = 1 cm.

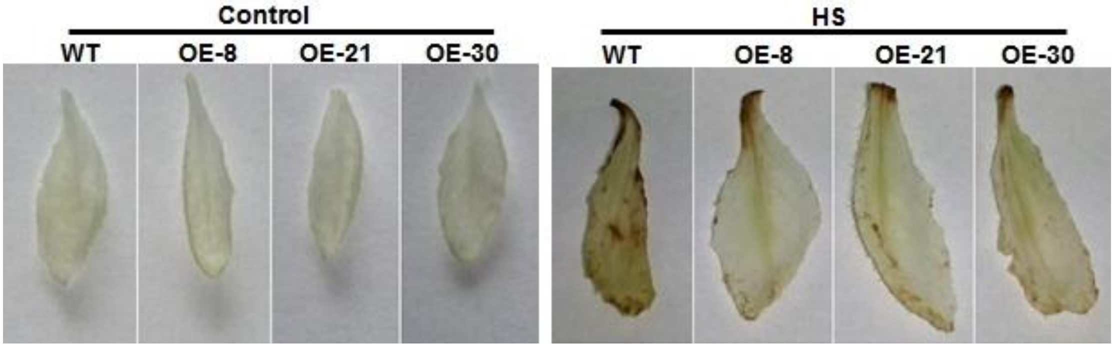

Figure S6. Histochemical detection of H_2O_2 with diaminobenzidine (DAB) staining in WT and transgenic *A. thaliana* rosette leaves under HS (45 ℃ for 4 h). WT: wild-type. OE-8, OE-21, and OE-30: transgenic *A. thaliana* plants.

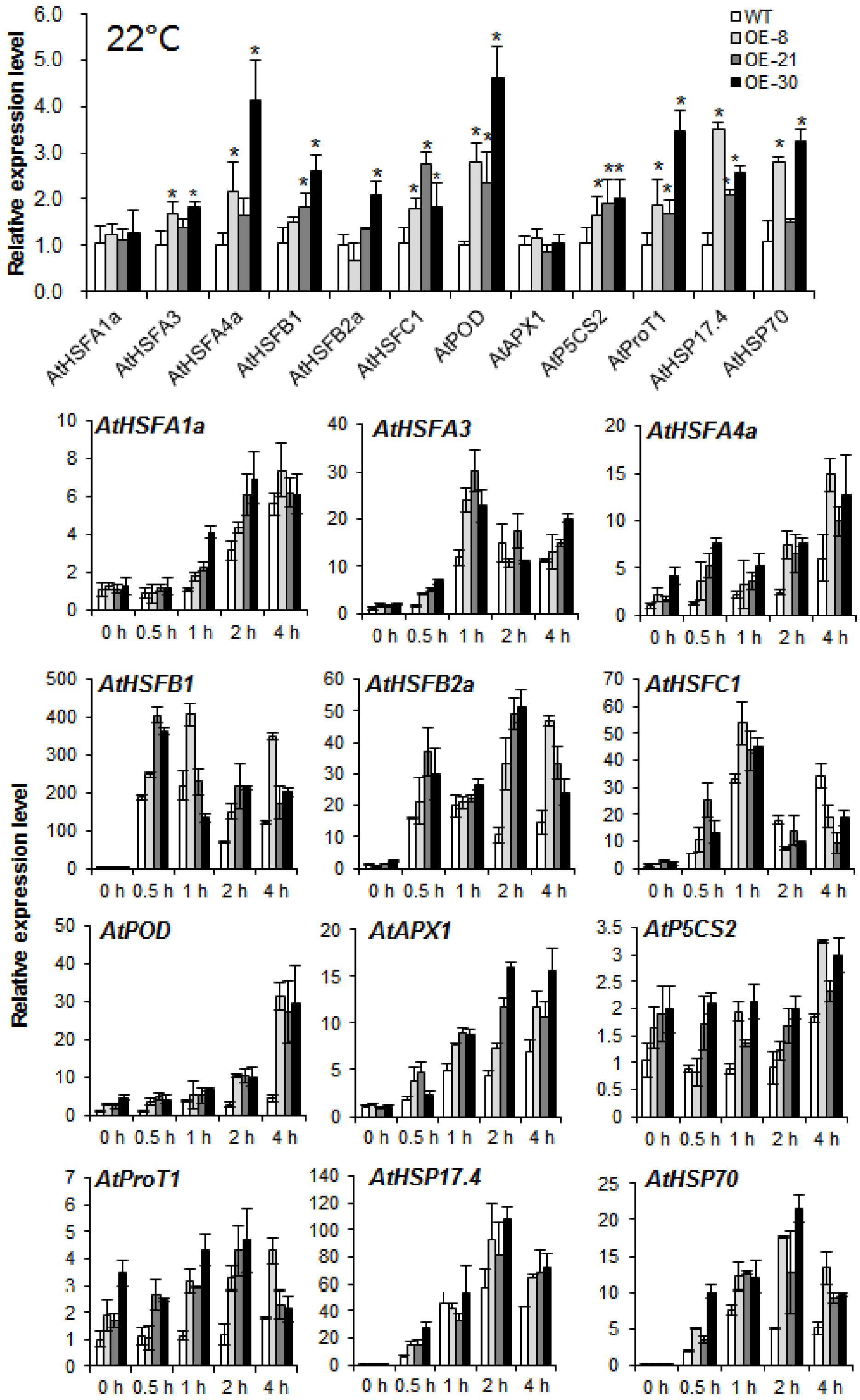

Figure S7. Expression analysis of HS-responsive genes in WT and transgenic *A. thaliana* plants. Data are the means ± standard deviations of three independent experiments. Significant differences between WT and transgenic *A. thaliana* plants are indicated by asterisks (*$P < 0.05$).

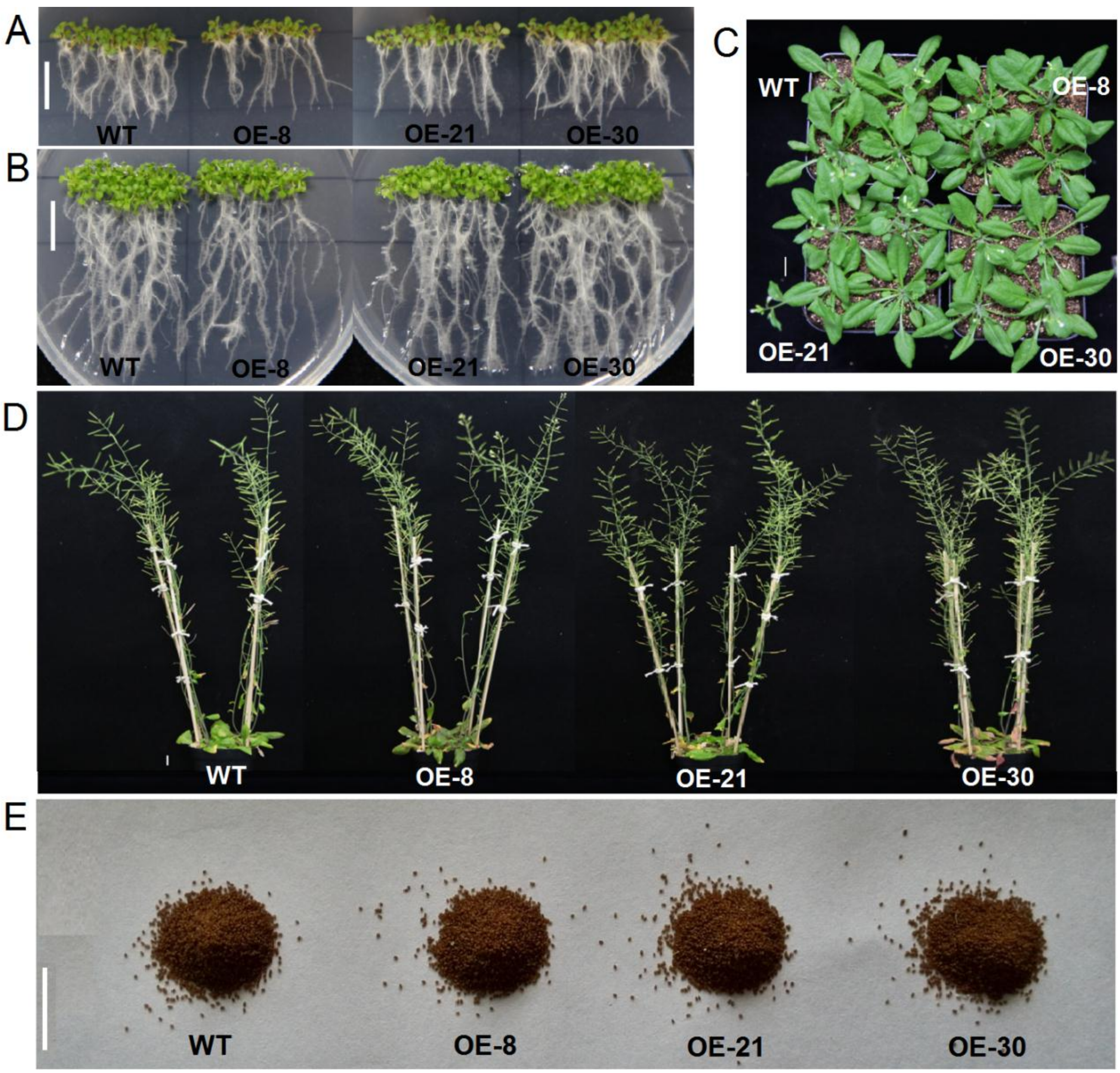

Figure S8. Phenotypes of *A. thaliana* under normal conditions. (**a**) 1-week-old and (**b**) 2-week-old plants grown on 1/2 MS medium. (**c**) 5-week-old and (**d**) 7-week-old seedlings cultivated in soil. (**e**) Seed yields per plant. *Scale bar* = 1 cm.

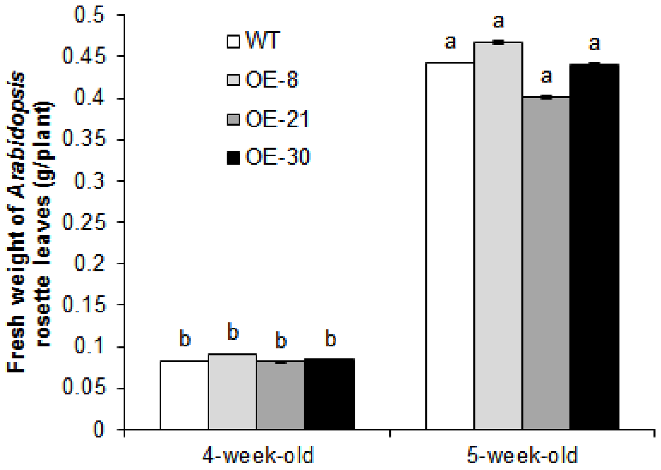

Figure S9. Fresh weights of *A. thaliana* rosette leaves (g/plant). The values are from four replicates of 40 seedlings (means ± standard deviations). Significant differences between WT and transgenic *A. thaliana* plants are indicated by different letters ($P < 0.05$).

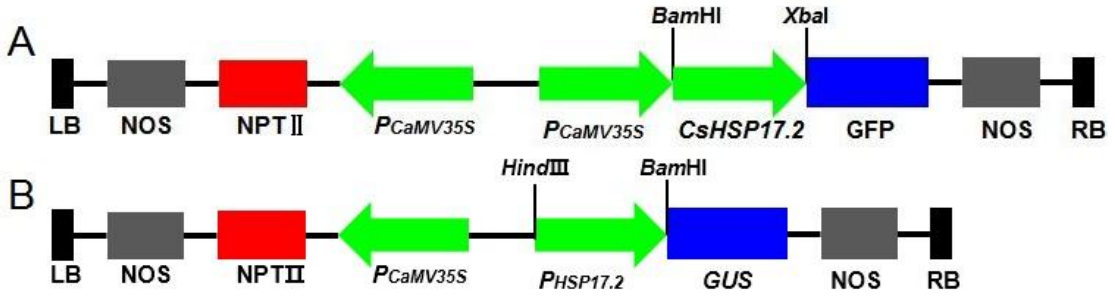

Figure S10. Structure of the *35S:CsHSP17.2:GFP* (a) and *$P_{HSP17.2}$:GUS* (b) recombinant vector used for *A. thaliana* transformations.

Table S1. Description of some *cis*-acting elements found in the *CsHSP17.2* promoter using the PlantCARE database.

Motif name	Sequence	Description
TATA-box	TAATA<br>GCTATAAATA	core promoter element around -30 of transcription start
HSE<br>(Heat Shock Element)	AGAAAGTTCG	*cis*-acting element involved in heat stress responsiveness
ARE<br>(Anaerobic Response Element)	AAACCA	*cis*-acting regulatory element essential for the anaerobic induction
MBS<br>(MYB Binding Site)	CAGTTG	MYB binding site involved in drought-inducibility
TC-rich repeats	ATTTACTCCA	*cis*-acting element involved in defense and stress responsiveness
GCN4_motif	TGACACA	*cis*-regulatory element involved in endosperm expression
O2-site	GATGATATGA	*cis*-acting regulatory element involved in zein metabolism regulation
Skn-1_motif	GTCAT	*cis*-acting regulatory element required for endosperm expression
CGTCA-motif	CGTCA	*cis*-acting regulatory element involved in the MeJA-responsiveness
ERE<br>(Ethylene-Responsive Element)	TTTGAAA	ethylene-responsive element probably involved in petal senescence
GARE-motif<br>P-box	TCTGTTG<br>CAAAAGG	gibberellin-responsive element
rGTbcS-CMA7a<br>G-box	GTCGATAAGC<br>CACGTC	*cis*-acting regulatory element involved in light responsiveness
CCAAT box	CCAAT<br>ATTGG	heat shock-related element
AGGGG motif	AGGGG	stress responsive element

Table S2. Primers used in this study.

**Primer name**	**Sequences (5′-3′)**	**Annotation**
ORF-F	ATGTCGATGATCCCAAGCTTCTTCGGC	ORF cloning
ORF-R	CTAACCAGAGATATCAATAGACTTGAT	
CsHSP17.2-qF	CCCTTCAGAAACTCTCCCTTC	qRT-PCR of *CsHSP17.2*
CsHSP17.2-qR	AACCCTGTGCCAAGTATCGT	
β-actin-F	GCCATCTTTGATTGGAATGG	Reference gene of *C. sinensis*
β-actin-R	GGTGCCACAACCTTGATCTT	
sub-F	CGGGATCCATGTCGATGATCCCAAGCTTC	Subcellular localization and *A.*
sub-R	GCTCTAGAACCAGAGATATCAATAGACTTG	*thaliana* transformation
pro-F	CGGGATCCATGTCGATGATCCCAAGCTTC	Overexpression in *E. coli* and *P.*
pro-R	CGGAATTCCTAACCAGAGATATCAATAGA	*pastoris*
S.tag	CGAACGCCAGCACATGGACA	Universal primers for pET-32a
T7-Ter	GCTAGTTATTGCTCAGCGG	(+) vector
5′AOX1	GACTGGTTCCAATTGACAAGC	Universal primers for pPIC3.5K
3′AOX1	GCAAATGGCATTCTGACATCC	vector
SP1	CCCTGTGCCAAGTATCGTTCTTCTCCT	Promoter cloning
SP2	GGGAGAGTTTCTGAAGGGGTCAAGAGAG	
SP3	AGGACTCTGTTTGGATACGGAGAA	
pHSP17.2-F	CCCAAGCTTCCTTCTCGAGTATGGTGTTTGG	Construction of $P_{HSP17.2}$:*GUS*
pHSP17.2-R	CGGGATCCGATATGCGATCGGAATTCAAGG	fusion vector
T-pHSP17.2-F	TAGAGCAATCAGGGAGGGTAAGC	Confirmation of transformants
T-pHSP17.2-R	ACACGGAAATGTTCTGCGACGAG	with $P_{HSP17.2}$:*GUS*
GUS-qF	AGAGCTGATAGCGCGTGACAAAAAC	qRT-PCR of *GUS* gene
GUS-qR	TGTGAGCGTCGCAGAACATTACATT	

Note: the underlines indicated the restriction sites.

Table S3. Primers used for qRT-PCR in transgenic *A. thaliana* overexpressing *CsHSP17.2*.

Gene name	Gene AGI No.	Primer name	Sequences (5′-3′)
AtAPX1	AT1G07890	F	CCTGATGCTACCAAGGGTTGT
		R	GACCTATCCTTGTGGCATCGTC
AtPOD	AT3G49120	F	CCAAACTCTTCGTGGACTATGC
		R	AACTCTTGGTCGCTCTGGAT
AtP5CS2	AT3G55610	F	GGGACAAGTTGTGGATGGAGAC
		R	TGGTACAAACCTCAAGGAACAC
AtProT1	AT2G39890	F	TTTAGTTTGATGTCTGTTGCTGC
		R	AGAAATAAACCTGCCTAAGAATA
AtHsp18.2	AT5G59720	F	AGCAACGAACAATGTCTCTCATTCCAAGC
		R	AACCTTGACTTCTTCCTTCTTCAGGCCTG
AtHSP70	AT3G12580	F	GTCGAAATCATCGCCAACG
		R	CGACTTGATTCTTGGCAGCA
AtACTIN2	AT3G18780	F	CTCCCGCTATGTATGTCGCC
		R	TTGGCACAGTGTGAGACACAC
AtHSFA1a	AT4G17750	F	AATGCCAGAGACTTCCCAGAT
		R	AATGGGCTTGGAGAGATGAAT
AtHSFA3	AT5G03720	F	CTTGGGGACTGACCGGAGCTAGCTTCGTAG
		R	GCTAGTGCTACTGCAGCAAGTTTGGTTG
AtHSFA4a	AT4G18880	F	ATGATTCTTCATCCGATTCTATCGTCTCTTGG
		R	TCATCATTCGCAAATTCCCATTGCTCAGG
AtHSFB1	AT4G36990	F	GGACCGGGATGAAAAGAATTA
		R	CACGCTGGTTTGAACAGTCTT
AtHSFB2a	AT5G62020	F	GACGCATCAAACAGTTGTTGCTCCTTCGTC
		R	TCAGTGGGCTGAGATCCGACGTAATTCGAC
AtHSFC1	AT3G24520	F	CTTCTCGCAACGAATCTTACCTGCTTATTTC
		R	CTTGACCGTACATCCCCCGCGCGTGTTTC
